# Individuals of high socioeconomic status are altruistic in sharing money but egoistic in sharing time

**DOI:** 10.1038/s41598-022-14800-y

**Published:** 2022-06-27

**Authors:** Ulf Liebe, Nicole Schwitter, Andreas Tutić

**Affiliations:** 1 Department of Sociology, University of Warwick, Coventry, CV4 7AL UK; 2 Department of Sociology, University of Bergen, Rosenbergsgaten 39, 5015 Bergen, Norway

**Keywords:** Psychology, Human behaviour

## Abstract

The questions of whether and how socioeconomic status (SES) predicts prosocial behavior have sparked an interest from different disciplines, yet experimental evidence is inconclusive. We embedded two types of dictator games in a web survey with 7772 participants from Germany, Poland, Sweden, and the US. Each participant was asked to split a sum of money and a fixed amount of time between themself and a recipient. While higher-SES individuals are more generous than lower-SES individuals in the money game, they are more egoistic in the time game. In addition, the SES of the recipient matters more in the money game than in the time game. These results point towards the relevancy of a situationally contingent social norm of redistribution in studying the relationship between SES and prosocial behavior.

## Introduction

Are the rich more selfish than the poor? This question of whether socioeconomic status (SES) groups differ regarding prosocial and ethical behavior is especially important for states following the Western model of combining democracy and market capitalism, since these rely on consensual forms of redistribution to correct extreme forms of social inequality.

Research on the relationship between SES and prosocial behavior has received heightened interest across the behavioral and social sciences in the last decade^1–5^. Initially, social-psychological arguments according to which higher-status and lower-status individuals differ in their social outlooks were put forward to explain findings according to which the former behave less charitably, less generously, and less helpfully than the latter^1,2^. As replication attempts failed^6,7^ and studies which demonstrate a positive relationship between SES and prosocial behavior^3–5,8–12^ amassed, scholars started to explore in what relevant ways SES groups might differ above and beyond their social outlooks.

One important aspect in this “complex mosaic”^4^ is that choice situations which at first sight appear identical might actually differ for individuals with different socioeconomic backgrounds. By definition, SES groups differ in their income, education, and occupational prestige^2^. As a consequence of this, prosocial behavior which involves the transfer of monetary resources is relatively cheap for higher-status individuals and relatively costly for lower-status individuals^3,4,8,9^. Hence, studies measuring prosocial behavior by some type of monetary transfer^9^ and in particular studies using incentivized economic games^1,3,8^ are prone to find a positive relationship between SES and prosocial behavior.

However, differences in the costliness of monetary transfers are not the only factor that might bias studies using monetary transfers^9^. We suggest that situations in which monetary transfers are involved potentially activate a social norm of redistribution which applies differently to individuals depending on their socio-economic background. In Western societies, the idea that higher-SES individuals carry a pronounced responsibility for society and should transfer monetary resources to lower-SES individuals is institutionalized on the macro level in progressive tax systems^13^. The norm of “noblesse oblige” also prevails on the micro level of everyday interactions, for instance in car-pooling arrangements between a boss and an employee, in which the boss is expected to bear a greater share of the costs^14^.

Against this background, we are interested in the question of whether the salience of the norm of redistribution moderates the relationship between SES and prosocial behavior. Since status groups differ regarding their monetary endowment but are equal in terms of their endowment with time, we reason that a norm of redistribution is more salient in situations involving money than time. Hence, we embedded two types of dictator games, a money dictator game (MDG) and a time dictator game (TDG), in a web survey with 7722 individuals from Germany, Poland, Sweden, and the US. Each participant took part in both games. In the MDG, participants were asked to split $15 between themselves and another participant, while in the TDG, they were asked to split 5 min during which they should fulfil a simple but tedious task. To make the relevancy of the norm of redistribution observable, participants were informed about recipients’ SES.

## Results

Across the four countries, in the MDG the overwhelming majority of participants shared their endowment (88% across all countries; 91% in Germany; 80% in Poland; 91% in Sweden, 89% in the US) and, on average, they gave around 41% (sd = 27.0) across all countries (41%, sd = 23.6, in Germany; 33%, sd = 28.4, in Poland; 46%, sd = 25.4, in Sweden; 43%, sd = 28.6, in the US). In the TDG, participants allocated both more often (93% across all countries; 94% in Germany; 84% in Poland; 96% in Sweden, 95% in the US) and higher shares on average (49%, sd = 26.8 across all countries; 50%, sd = 23.9, in Germany; 42%, sd = 31.1, in Poland; 52%, sd = 22.5 in Sweden; 53%, sd = 27.7, in the US).

**Figure 1:**
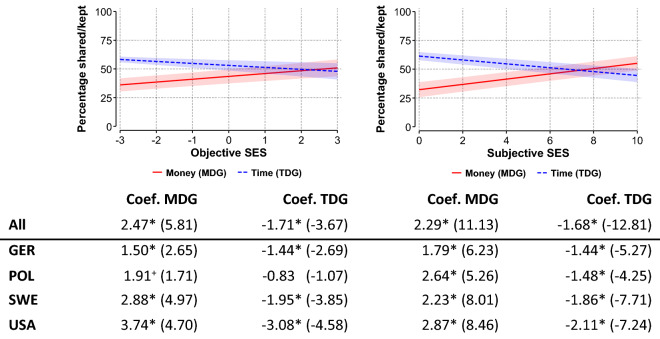
Higher-status individuals give more in the MDG and spend less time on the task in the TDG. Shown are results of Cragg models for the second stage on percentages shared in the MDG and percentages of work time kept in the TDG (y-axis), depending on objective SES (x-axis, left) and subjective SES (x-axis, right); shown are predicted percentages with 95% confidence intervals. The plots refer to all countries (pooled model), and coefficients and z-values of status effects are presented for all countries and separately for each country (Germany, Poland, Sweden, USA). For both MDG and TDG, higher values on the y-axis indicate more prosocial behavior. For both objective SES and subjective SES, higher values on the x-axis indicate higher SES. ^+^ and * denote significant differences at least at the 10% level and 5% level, respectively (for full models see Tables S1 and S2, suppl. material).

Figure 1 (left) shows for all four countries that monetary donations in percent of the endowment increase with objective SES, measured as a composite of education, income, and job prestige. By contrast, the time spent on fulfilling the task in percent decreases with SES. This result holds in all four countries and in a replication using a subjective measure of social status, the MacArthur scale, where participants compare themselves to their country’s society (Fig. 1, right). Except for objective SES effects in Poland (with *p* < 0.1 for the MDG and *p* > 0.1 for the TDG), all effects are significant (with *p* < 0.05). Therefore, compared to lower-status individuals, higher-status individuals act more altruistic in the MDG and less altruistic in the TDG.

**Figure 2:**
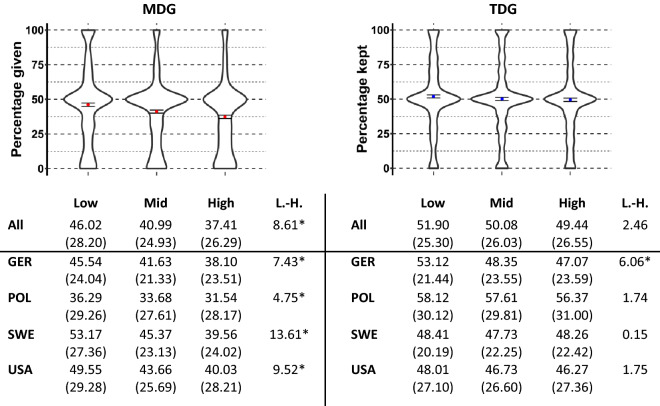
The redistribution across SES groups is more heterogenous in the MDG than in the TDG. Shown are violin plots of percentages given (MDG, y-axis left) and kept (TDG, y-axis right) with mean value and 95% confidence interval per SES of recipients (low, mid, high) in the MDG and TDG across all countries (pooled model), as well as mean values and standard deviations of percentages given (MDG) and kept (TDG) and the difference in mean values between low- and high-status recipients (L.-H.) for all countries and separately for each country (Germany, Poland, Sweden, USA); * denotes significant differences at least at the 5% level based on a two-tailed t-test for country models and bivariate regressions with cluster-robust standard errors for pooled models (see also Table S3, suppl. material).

As we varied the SES of the recipient in the MDG and TDG (next to a control group without any status information), we can test to what extent recipients’ status and hence the salience of the norm of redistribution can explain behavioral differences in both games. Figure 2 presents differences in distributions and mean values of donations depending on the SES of the recipient. Differences in mean values between low-status and high-status recipients range between 4.75 (Poland) and 13.61 (Sweden) percentage points in the MDG and 0.15 (Sweden) and 6.05 (Germany) percentage points in the TDG. In the MDG, we find strong recipient effects in all four countries: individuals donate considerably less to recipients with higher SES compared to recipients with lower SES (differences between high and low SES recipients are all significant with *p* < 0.05). Variations in distributions across the SES groups of recipients in the TDG are less notable (differences between high and low SES recipients are all insignificant with *p* > 0.1, except Germany with *p* < 0.05).

Tables S18 (MDG) and S19 (TDG) show Cragg hurdle models including interaction effects between status of participant and status of recipient. In the pooled model (models 1 and 2), we find that recipients of middle or high status receive less in the MDG the higher the status of respondents (both subjective and objective, all interaction effects are significant at least at the 10% level). Across countries, this pattern holds descriptively (except for Poland in case of the interaction between high-status recipient and objective social status), but significance level varies. The negative effect is not stronger for high-status respondents compared to middle-status respondents. Interaction effects are generally close to zero and not significant in the TDG (except for Germany in case of the interaction effect between high-status recipient and subjective social status, with *p* < 0.10).

## Discussion

Our results indicate that higher SES is associated with more altruism when distributing monetary endowments and more egoism when distributing time. By varying the SES of the recipient in the money and time experiments, we also uncovered that the status of recipients matters more for monetary compared with time transfers and that there was a redistribution from higher status individuals to lower status recipients for monetary transfers.

These findings support the idea that choice situations interact with individuals’ SES background in activating a social norm of redistribution. Since our study is based on specific monetary and time endowments, it remains an open question whether stake sizes matter and to what extent socioeconomic status groups differ in their evaluation of time and money. Note that our findings regarding the effects of the status of participants who acted in the role of the dictator can potentially be explained by the assumption that higher-status actors have a rather low valuation of money and a rather high valuation of time in comparison to lower-status actors. However, this assumption cannot necessarily explain our findings regarding the effects of the status of the recipients. In contrast, the situationally contingent activation of a norm of redistribution can explain both types of status effects observed in our study.

Future research is also needed to assess whether a social norm of redistribution plays a role in other choice situations used to study the interplay of SES and prosocial behavior and to what extent its influence stretches beyond Western societies. In particular, non-reactive field experiments are needed to check whether the norm of redistribution is also activated if potential experimenter demand effects and social desirability bias are excluded^9^.

Another promising avenue for future research is to link our findings with research on the moderating role of economic inequality on the status-prosociality nexus^15–17^. In a landmark study using data from the US, Côté et al. have demonstrated that in American states with high levels of economic inequality higher status actors are less generous with money than lower status actors, whereas the relationship between status and generosity is reversed in American states with less economic inequality^15^. However, other studies relying mostly on between-country comparisons could not replicate this finding^16,17^. Since the salience of a norm of redistribution might be influenced by the prevailing economic conditions as well as cultural context factors related to the tolerance of inequality, the situationally contingent activation of a norm of redistribution might also help in explaining this conflicting evidence.

Speaking generally from a sociological point of view, the situationally contingent activation of social norms is an important determinant of prosocial behavior^18^. Since certain norms apply differentially to different status groups, they add a further layer of complexity to the “complex mosaic”^4^ of the status-prosociality nexus.

## Methods

This study was conducted with approval from the Humanities and Social Sciences Research Ethics Committee of the University of Warwick (reference number: HSSREC 111/19-20) and all methods were performed in accordance with its relevant guidelines and regulations. All participants provided informed consent to take part in this study.

This study is based on a web survey for which the survey firm Respondi sampled adult respondents from four different countries: USA, Germany, Poland, Sweden. Respondi uses both online channels and telephone interviews to recruit members of their access panel. The main survey was in the field from April 2021 to June 2021 following a pretest phase with 100 respondents per country. We employed simple quotas on education, age, and gender in line with population characteristics of the respective countries. Our final sample includes 8000 respondents (2000 per country) of which 7828 participated in the MDG and 7785 participated in the TDG; 7722 respondents participated in both games.

**Figure 3:**
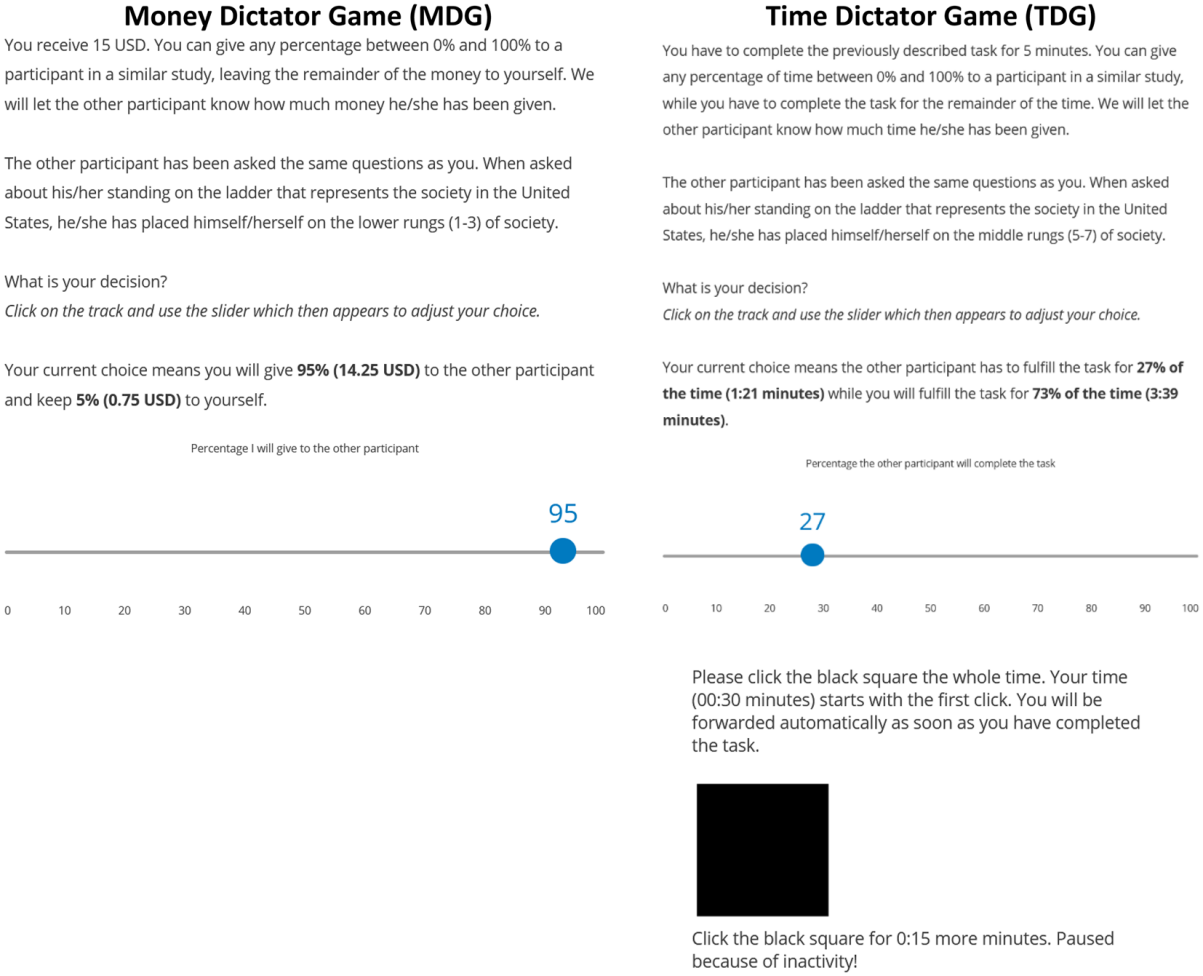
Experimental setups of the MDG (left) and TDG (right) with status information on recipients. The measure of the TDG was reverse coded in the analysis for higher values to express more prosociality.

In both the time and the money setup, we implemented four different wordings of the MDG and the TDG and assigned participants randomly to one of the four alternatives. The wordings differed in the way the other player, the recipient of the money/time, was described: Participants were either splitting the time or money with a respondent with a socially low (Scale points 1–3), middle (Scale points 5–7) or high (Scale points 9–11) standing according to the MacArthur ladder, or they did not receive any status information at all (see Fig. 3). The experiments conducted do not involve deception, and 100 primary participants from each country were randomly selected to get paid out according to their decisions in the MDG. One hundred further participants of the same country and matching in subjective social status as presented in the experiment were selected and received the share of the money the primary respondent decided to share. Similarly, one hundred random participants were also selected to conduct the assigned share of the time task. Before answering the experimental tasks, all participants were informed about this procedure. All respondents additionally received a reimbursement for completing the survey.

We used both objective and subjective measures of social class. We have constructed a composite measure of objective social status, equaling the standardized average of respondents’ education (ISCED), occupational prestige (SIOPS), and the decile of the household OECD-equivalized income. We first z-standardized our measures of education, occupational prestige and income within each country. Following this, we calculate a simple arithmetic mean, which we then again standardize within each country.

Missing values for income (n = 159; 2% of all observations) and job prestige (n = 3889, excluding Sweden; 64.8% of the observations) were imputed using stochastic regression separately for each country. Income was regressed on age, gender, education, employment situation, and household size; the regression model of job prestige additionally included income. According to the R^2^, the imputation model for income explains between 4% (Poland) and 23% (USA) of the variance, while that for job prestige explains between 25% (Poland) and 32% (Germany). The composite measure of social status for Sweden does not include job prestige, as the automatic assignment of ISCO codes and SIOPS values to the jobs (using the EurOccupations Database) was not possible. We re-run our analyses for all countries and all dimensions of objective SES (both with and without imputed values) to check for robustness of our findings irrespective of status indicator and imputation. In most instances, there is a positive relationship between status indicator and money giving and a negative relationship between status indicator and time giving irrespective of the indicator used; models and further clarifications can be found in the supplementary material (see models in Tables S3–S13). Subjective socioeconomic status was measured using the McArthur Scale. Respondents were asked to place themselves on an 11-rung ladder representing their country’s society. For descriptives on samples and status variables see Table 1.

**Table 1:** Descriptives of sample and status variables (mean, standard deviation, minimum/maximum).

	Germany	Poland	Sweden	US
Composite objective status (cent.)	0.0041	0.0088	0.017	− 0.0045
	1.00	0.99	0.98	1.00
	− 3.04/3.14	− 3.12/2.69	− 2.87/2.21	− 3.04/2.99
Subjective socio-economic status	5.02	5.00	5.02	4.70
	1.95	2.10	2.10	2.33
	0/10	0/10	0/10	0/10
Education (ISCED)	3.49	3.66	3.69	3.66
	1.08	1.02	1.22	1.22
	0/6	0/6	0/6	0/6
Income deciles	5.46	5.45	5.51	5.47
	2.84	2.85	2.85	2.89
	1/10	1/10	1/10	1/10
Job prestige (SIOPS)	42.50	43.06	Not available	42.57
	14.03	13.91		13.87
	12.00/92.35	12.00/86.47		12.00/98.01
Age	49.34	42.29	48.91	45.47
	16.84	14.88	18.04	17.74
	18/87	18/85	18/90	18/95
Gender (male)	0.490	0.477	0.481	0.495
	0/1	0/1	0/1	0/1
Number of observations	1962	1924	1911	1925

No standard deviation is given for binary variables.

Data was analyzed using Cragg hurdle models, two-part models which combine a selection model with an outcome model and thus separate the decisions of sharing money or time into two-step processes. It comprises separate estimates of a binary probit model and a truncated regression. For each country, we run four different models. We regress the percentage given in the MDG and the percentage kept in the TDG on either subjective social status or objective social status, and control for the status of the receiver, as well as age and gender of respondents. The main models can be found in the supplementary material (Tables S1 and S2). Models including interaction effects between status of participant and status of recipient are presented in Tables S18 and S19 in the supplementary material.

For each of the 20 models (16 country models, 4 pooled models), we compare the Cragg hurdle models to standard OLS which is outperformed in all cases according to Akaike’s and Schwarz’s Bayesian information criteria. For example, for the pooled model (comparing the Cragg hurdle models 1/2 in Table S1 with models 1/2 in Table S16, as well as models 11/12 in Table S2 with models 11/12 in Table S17), the absolute difference in AIC is 5363 using objective SES (5368 using subjective SES) in the MDG, and 2964 using objective SES (2956 using subjective SES) in the TDG. The absolute difference in BIC is 5356 using objective SES (5361 using subjective SES) in the MDG, and 2957 using objective SES (2949 using subjective SES) in the TDG. All standard OLS model results can be found in the supplementary material (Tables S16 and S17) and can be compared with the main Cragg hurdle models (Tables S1 and S2).

We further checked whether seemingly unrelated regression equations models (SUR) would affect our significance levels; this was the case for Poland in the MDG (with *p* = 0.10), but not in any other models. SUR allow the error terms in two different models to correlate. Parameter estimates and associated (co)variance matrices are combined into one parameter vector and simultaneous (co)variance matrix of the sandwich/robust type. This considers that the same respondents were playing both the MDG and the TDG. The results can be found in the supplementary material (Tables S14 and S15).

Supplementary Materials contain more information regarding statistical analyses and all aforementioned models. Data and code can be found on GitHub (https://github.com/nschwitter/MDGTDG).

## Supplementary Information

Supplementary Information.
