# Supplementary material for: Individuals of high socioeconomic status are altruistic in sharing money but egoistic in sharing time
Source: Sci Rep. 2022 Jun 27;12:10831. doi: 10.1038/s41598-022-14800-y (PMC9237058; doi:10.1038/s41598-022-14800-y)
Supplement: Supplementary file 1 — Supplementary Information. [file 41598_2022_14800_MOESM1_ESM.docx]

**Supplementary Material**

**Individuals of high socioeconomic status are altruistic in sharing money**

**but egoistic in sharing time**

Ulf Liebe, Nicole Schwitter, Andreas Tutic*

*Corresponding author. Email: andreas.tutic@uib.no

**This file includes:**

Supplementary Text

Tables S1 to S19

Supplementary Text

Measurement of Subjective Status

Subjective socioeconomic status was measured using the McArthur Scale. Respondents were asked to place themselves on an 11-rung ladder representing their country’s society (“Think of a ladder as representing where people stand in the [country]. At the top of the ladder (11) are the people who are the best off – those who have the most money, most education and best jobs. At the bottom (1) are the people who are the worst off – who have the least money, least education and the worst jobs or no jobs. The higher up you are on this ladder, the closer you are to people at the very top and the lower you are, the closer you are to people at the very bottom. Where would you place yourself on this ladder?”).

Statistical Analyses

In the following, we present a number of tables. Table S1 and S2 are the main Cragg Hurdle models on the percentage of money shared in the MDG (S1) and on the percentage of time kept in the TDG (S2), underlying Figure 1. The first two columns are pooled models, the other columns are country-models. Due to four missing values in the subjective SES, models using it have slightly fewer observations. Table S3 shows recipient effects (underlying Figure 2).

Tables S4 to S13 provide additional robustness checks. Tables S4-S8 provide robustness checks for the MDG, Tables S9-S13 provide robustness checks for the TDG. We present one table for the pooled dataset and one table for each of the four countries. We run up to 10 different models per table. In model M1, we remove the top and bottom 5% of observations regarding objective SES. In M2-M6, we use separate indicators (for job prestige and income, we show effects when using all observations or only cases without imputation) of objective SES instead of the composite measure. M7-M10 show the effect of different composite measures. This allows us to check whether the results are driven by outliers or specific status dimensions. Replication materials are also on Github (https://github.com/nschwitter/MDGTDG).

Overall, the main results of our paper are relatively stable across the different model specifications. For the MDG, we find significant positive effects of all measures of SES in the pooled model, and in Sweden; in Germany and the US, all measures except job prestige (including imputed values in Germany, excluding imputed values in the US) are positive and significant. In Poland, we find effects of job prestige (both imputed and not imputed) and the composite measures which includes all measures (10% significance level), and the composite measure including income and job prestige (5% significance level).

In the TDG, we find significant negative effects of all measures of SES in Sweden. In the pooled model and in Germany, income has a negative but non-significant effect; in Germany, job prestige excluding imputed values does not show a significant negative effect. While we did not report a significant and negative effect for the composite measure in Poland, we find significant (at least 5% level) negative effects of education and job prestige, as well as the composite measure which does not include income.

All results refer to the second stage in the Cragg models. At the first stage, measures of objective social status do not seem to play a role when deciding whether to give any money/time at all.

Tables S14 (MDG) and S15 (TDG) show seemingly unrelated regression equations where the error terms in the corresponding MDG and TDG equation were allowed to correlate. This affected the significance level of objective SES on the MDG in Poland (with p=0.10 instead of previously p<0.10), but not in any of the other models.

Table S16 (MDG) and S17 (TDG) show standard OLS results of the main models. The Cragg hurdle models (Tables S1 and S2) outperform standard OLS in all cases according to Akaike’s and Schwarz’s Bayesian information criteria.

Tables S18 (MDG) and S19 (TDG) show Cragg Hurdle models including interaction effects between status of participant and status of recipient. In the pooled model (models 1 and 2), we find that recipients of middle or high status receive less in the MDG the higher the status of respondents (both subjective and objective, all interaction effects are significant at least at the 10% level). Across countries, this pattern holds descriptively (except for Poland in case of the interaction between high-status recipient and objective social status), but significance level varies. The negative effect is not stronger for high-status respondents compared to middle-status respondents. Interaction effects are generally close to zero and not significant in the TDG (except for Germany in case of the interaction effect between high-status recipient and subjective social status, with p<0.10).

**Table S1.** Main Cragg Hurdle Models on the percentage of money shared in the MDG, underlying Figure 1 in the main text

|  | (1)  Pooled model | (2)  Pooled model | (3)  Germany | (4) Germany | (5)  Poland | (6)  Poland | (7)  Sweden | (8)  Sweden | (9)  USA | (10)  USA |
| --- | --- | --- | --- | --- | --- | --- | --- | --- | --- | --- |
|  | Objective SES | Subjective SES | Objective SES | Subjective SES | Objective SES | Subjective SES | Objective SES | Subjective SES | Objective SES | Subjective SES |
| Percentage given MDG |  |  |  |  |  |  |  |  |  |  |
| Social status (composite, cent.) | 2.473*** (5.81) |  | 1.496** (2.65) |  | 1.906+ (1.71) |  | 2.878*** (4.97) |  | 3.740*** (4.70) |  |
|  |  |  |  |  |  |  |  |  |  |  |
| Subjective social status |  | 2.290*** (11.13) |  | 1.785^***^ (6.23) |  | 2.637^***^ (5.26) |  | 2.232^***^ (8.01) |  | 2.871^***^ (8.46) |
|  |  |  |  |  |  |  |  |  |  |  |
| Recipient: No status info | -6.736*** (-4.72) | -6.609*** (-4.00) | -5.180*** (-3.31) | -4.689^**^ (-3.03) | -5.271+ (-1.79) | -5.268+ (-1.83) | -5.898*** (-3.73) | -5.704^***^ (-3.66) | -11.15*** (-5.05) | -11.84^***^ (-5.48) |
|  |  |  |  |  |  |  |  |  |  |  |
| Recipient: Middle status | -5.167*** (-7.71) | -5.303*** (-7.28) | -4.036** (-2.65) | -4.077^**^ (-2.71) | -4.248 (-1.47) | -4.329 (-1.53) | -6.412*** (-4.11) | -6.355^***^ (-4.13) | -6.323** (-2.95) | -6.974^***^ (-3.32) |
|  |  |  |  |  |  |  |  |  |  |  |
| Recipient: High status | -9.151*** (-5.68) | -9.187*** (-5.68) | -7.020*** (-4.49) | -6.748^***^ (-4.36) | -5.994* (-2.03) | -6.444^*^ (-2.24) | -13.07*** (-8.20) | -13.01^***^ (-8.29) | -9.984*** (-4.54) | -10.28^***^ (-4.78) |
|  |  |  |  |  |  |  |  |  |  |  |
| Age | 0.00777 (0.16) | 0.00795 (0.15) | 0.00382 (0.12) | -0.00354 (-0.11) | 0.0683 (0.92) | 0.100 (1.46) | 0.0273 (0.81) | 0.0161 (0.48) | -0.110* (-2.47) | -0.100^*^ (-2.33) |
|  |  |  |  |  |  |  |  |  |  |  |
| Male | 2.909* (2.27) | 2.533+ (1.80) | 0.902 (0.81) | 0.778 (0.71) | 6.744** (3.15) | 7.078^***^ (3.41) | 0.763 (0.62) | -0.0501 (-0.04) | 4.928** (3.13) | 3.901^*^ (2.55) |
|  |  |  |  |  |  |  |  |  |  |  |
| Constant | 46.89*** (8.91) | 35.78*** (6.16) | 46.96*** (23.14) | 38.26^***^ (15.30) | 29.27*** (7.30) | 14.92^**^ (3.23) | 53.99*** (28.90) | 43.62^***^ (19.27) | 53.88*** (20.56) | 40.81^***^ (13.74) |
| Given MDG > 0 |  |  |  |  |  |  |  |  |  |  |
| Social status (composite, cent.) | 0.0153 (1.07) |  | 0.0273 (0.68) |  | 0.0196 (0.56) |  | 0.0522 (1.22) |  | -0.0259 (-0.67) |  |
|  |  |  |  |  |  |  |  |  |  |  |
| Subjective social status |  | 0.0371* (2.40) |  | 0.0260 (1.27) |  | 0.0161 (1.04) |  | 0.0871^***^ (4.46) |  | 0.0422^**^ (2.62) |
|  |  |  |  |  |  |  |  |  |  |  |
| Recipient: No status info | -0.303*** (-9.38) | -0.300*** (-8.70) | -0.349** (-3.00) | -0.341^**^ (-2.93) | -0.268** (-2.93) | -0.268^**^ (-2.92) | -0.236+ (-1.86) | -0.222+ (-1.74) | -0.384*** (-3.47) | -0.400^***^ (-3.59) |
|  |  |  |  |  |  |  |  |  |  |  |
| Recipient: Middle status | -0.112* (-1.96) | -0.113* (-1.98) | -0.0681 (-0.55) | -0.0681 (-0.55) | -0.0393 (-0.41) | -0.0392 (-0.41) | -0.305* (-2.47) | -0.296^*^ (-2.38) | -0.127 (-1.09) | -0.142 (-1.21) |
|  |  |  |  |  |  |  |  |  |  |  |
| Recipient: High status | -0.242*** (-4.41) | -0.240*** (-4.47) | -0.252* (-2.11) | -0.237^*^ (-1.98) | -0.144 (-1.53) | -0.147 (-1.57) | -0.370** (-3.03) | -0.360^**^ (-2.92) | -0.298** (-2.63) | -0.314^**^ (-2.77) |
|  |  |  |  |  |  |  |  |  |  |  |
| Age | 0.00891*** (9.04) | 0.00892*** (8.36) | 0.00689** (2.78) | 0.00700^**^ (2.84) | 0.00596* (2.54) | 0.00637^**^ (2.85) | 0.0101*** (3.95) | 0.00953^***^ (3.66) | 0.00653** (2.94) | 0.00613^**^ (2.77) |
|  |  |  |  |  |  |  |  |  |  |  |
| Male | -0.0862* (-2.11) | -0.0947* (-2.04) | -0.142+ (-1.73) | -0.149+ (-1.81) | 0.00641 (0.10) | 0.00912 (0.14) | -0.140 (-1.53) | -0.192^*^ (-2.08) | -0.153* (-2.00) | -0.151^*^ (-1.97) |
|  |  |  |  |  |  |  |  |  |  |  |
| Constant | 0.980*** (5.17) | 0.805*** (5.26) | 1.284*** (8.42) | 1.152^***^ (6.17) | 0.702*** (5.86) | 0.603^***^ (4.39) | 1.198*** (8.52) | 0.826^***^ (5.08) | 1.222*** (9.17) | 1.058^***^ (7.16) |
| lnsigma |  |  |  |  |  |  |  |  |  |  |
| Constant | 3.265*** (36.47) | 3.251*** (36.68) | 3.092^***^ (151.00) | 3.082^***^ (151.25) | 3.479^***^ (109.53) | 3.461^***^ (110.54) | 3.113^***^ (157.84) | 3.100^***^ (158.00) | 3.352^***^ (140.24) | 3.333^***^ (141.24) |
| Observations | 7722 | 7718 | 1962 | 1961 | 1924 | 1922 | 1911 | 1910 | 1925 | 1925 |
| Log likelihood | -33628.6 | -33529.6 | -8504.9 | -8485.7 | -7980.3 | -7953.0 | -8359.5 | -8324.7 | -8534.1 | -8505.9 |
| AIC | 67266.26 | 67067.25 | 17039.88 | 17001.45 | 15990.5 | 15935.92 | 16748.98 | 16679.49 | 17098.22 | 17041.73 |
| BIC | 67294.07 | 67095.06 | 17123.61 | 17085.17 | 16073.93 | 16019.34 | 16832.31 | 16762.81 | 17181.66 | 17125.17 |

Note: z statistics in parentheses; + p<0.10, * p<0.05, ** p<0.01, *** p<0.001.

**Table S2.** Main Cragg Hurdle Models on the percentage of time kept in the TDG, underlying Figure 1 in the main text

|  | (11)  Pooled model | (12)  Pooled model | (13)  Germany | (14) Germany | (15)  Poland | (16) Poland | (17)  Sweden | (18) Sweden | (19)  USA | (20)  USA |
| --- | --- | --- | --- | --- | --- | --- | --- | --- | --- | --- |
|  | Objective  SES | Subjective  SES | Objective  SES | Subjective SES | Objective  SES | Subjective SES | Objective  SES | Subjective SES | Objective  SES | Subjective SES |
| Percentage kept TDG |  |  |  |  |  |  |  |  |  |  |
| Social status (composite, cent.) | -1.713*** (-3.67) |  | -1.444** (-2.69) |  | -0.825 (-1.07) |  | -1.954*** (-3.85) |  | -3.084*** (-4.58) |  |
|  |  |  |  |  |  |  |  |  |  |  |
| Subjective social status |  | -1.677*** (-12.81) |  | -1.442^***^ (-5.27) |  | -1.483^***^ (-4.25) |  | -1.857^***^ (-7.71) |  | -2.108^***^ (-7.24) |
|  |  |  |  |  |  |  |  |  |  |  |
| Recipient: No status info | 0.813 (1.13) | 0.631 (0.81) | -0.999 (-0.67) | -1.297 (-0.87) | 1.646 (0.81) | 1.505 (0.75) | 1.189 (0.85) | 1.120 (0.81) | 0.791 (0.42) | 0.675 (0.37) |
|  |  |  |  |  |  |  |  |  |  |  |
| Recipient: Middle status | -1.271 (-1.07) | -1.256 (-1.04) | -3.801* (-2.54) | -3.838^*^ (-2.58) | -1.510 (-0.74) | -1.395 (-0.69) | 1.628 (1.18) | 1.650 (1.22) | -1.808 (-0.97) | -1.889 (-1.02) |
|  |  |  |  |  |  |  |  |  |  |  |
| Recipient: High status | -2.389 (-1.60) | -2.285 (-1.51) | -5.732*** (-3.88) | -5.534^***^ (-3.77) | -2.203 (-1.08) | -2.260 (-1.12) | 0.945 (0.68) | 1.297 (0.95) | -2.356 (-1.25) | -2.578 (-1.38) |
|  |  |  |  |  |  |  |  |  |  |  |
| Age | -0.106*** (-3.87) | -0.105*** (-3.34) | -0.0489 (-1.54) | -0.0408 (-1.30) | -0.0399 (-0.79) | -0.0530 (-1.11) | -0.116*** (-3.91) | -0.104^***^ (-3.52) | -0.0615 (-1.64) | -0.0700+ (-1.90) |
|  |  |  |  |  |  |  |  |  |  |  |
| Male | -0.717 (-0.89) | -0.439 (-0.45) | -0.0247 (-0.02) | 0.0869 (0.08) | -2.848+ (-1.95) | -2.884^*^ (-2.00) | 1.216 (1.12) | 2.048+ (1.91) | -1.737 (-1.30) | -1.043 (-0.79) |
|  |  |  |  |  |  |  |  |  |  |  |
| Constant | 59.08*** (17.03) | 67.15*** (19.91) | 56.73*** (29.35) | 63.53^***^ (26.99) | 64.51*** (24.74) | 72.47^***^ (24.10) | 54.21*** (32.67) | 62.36^***^ (32.37) | 54.66*** (24.77) | 64.71^***^ (25.91) |
| Kept TDG > 0 |  |  |  |  |  |  |  |  |  |  |
| Social status (composite, cent.) | -0.00682 (-0.19) |  | 0.0490 (0.98) |  | 0.0169 (0.37) |  | 0.0292 (0.58) |  | -0.0928* (-2.28) |  |
|  |  |  |  |  |  |  |  |  |  |  |
| Subjective social status |  | -0.0307** (-2.59) |  | 0.00996 (0.41) |  | -0.0481^*^ (-2.43) |  | -0.0238 (-1.04) |  | -0.0495^**^ (-2.96) |
|  |  |  |  |  |  |  |  |  |  |  |
| Recipient: No status info | -0.253** (-3.13) | -0.256** (-3.18) | -0.360* (-2.48) | -0.359^*^ (-2.47) | -0.143 (-1.23) | -0.154 (-1.31) | -0.532*** (-3.52) | -0.534^***^ (-3.53) | -0.151 (-1.37) | -0.147 (-1.34) |
|  |  |  |  |  |  |  |  |  |  |  |
| Recipient: Middle status | -0.108 (-0.92) | -0.111 (-0.97) | -0.300* (-2.02) | -0.301^*^ (-2.03) | 0.0994 (0.79) | 0.0966 (0.76) | -0.448** (-2.95) | -0.448^**^ (-2.94) | 0.0207 (0.18) | 0.0143 (0.12) |
|  |  |  |  |  |  |  |  |  |  |  |
| Recipient: High status | -0.0576 (-0.84) | -0.0592 (-0.89) | -0.211 (-1.41) | -0.214 (-1.43) | 0.0256 (0.21) | 0.00829 (0.07) | -0.256 (-1.60) | -0.250 (-1.56) | 0.0444 (0.38) | 0.0395 (0.34) |
|  |  |  |  |  |  |  |  |  |  |  |
| Age | 0.00331*** (4.05) | 0.00335*** (3.76) | -0.000422 (-0.14) | -0.000678 (-0.23) | 0.00286 (0.92) | 0.00307 (1.03) | 0.00433 (1.49) | 0.00457 (1.56) | 0.00269 (1.15) | 0.00236 (1.01) |
|  |  |  |  |  |  |  |  |  |  |  |
| Male | -0.209*** (-5.35) | -0.206*** (-5.41) | -0.325** (-3.24) | -0.324^**^ (-3.24) | -0.127 (-1.47) | -0.123 (-1.42) | -0.264* (-2.46) | -0.239^*^ (-2.22) | -0.206* (-2.55) | -0.192^*^ (-2.38) |
|  |  |  |  |  |  |  |  |  |  |  |
| Constant | 1.566*** (9.47) | 1.722*** (17.11) | 2.094*** (10.87) | 2.055^***^ (9.05) | 1.391*** (8.79) | 1.643^***^ (8.99) | 1.904*** (10.50) | 2.001^***^ (9.59) | 1.337*** (9.75) | 1.583^***^ (10.06) |
| lnsigma |  |  |  |  |  |  |  |  |  |  |
| Constant | 3.221*** (37.22) | 3.213*** (36.55) | 3.100^***^ (167.28) | 3.094^***^ (167.22) | 3.360^***^ (169.23) | 3.354^***^ (169.34) | 3.020^***^ (163.90) | 3.006^***^ (163.73) | 3.254^***^ (155.04) | 3.244^***^ (155.35) |
| Observations | 7722 | 7718 | 1962 | 1961 | 1924 | 1922 | 1911 | 1910 | 1925 | 1925 |
| Log likelihood | -34844.9 | -34775.7 | -8728.9 | -8714.7 | -8898.4 | -8882.0 | -8363.3 | -8334.7 | -8589.1 | -8571.6 |
| AIC | 69697.72 | 69559.42 | 17487.75 | 17459.35 | 17826.84 | 17793.96 | 16756.64 | 16699.31 | 17208.18 | 17173.26 |
| BIC | 69725.53 | 69587.23 | 17571.47 | 17543.07 | 17910.27 | 17877.38 | 16839.97 | 16782.63 | 17291.62 | 17256.70 |

Note: z statistics in parentheses; + p<0.10, * p<0.05, ** p<0.01, *** p<0.001.

**Table S3.** Recipient effects and t-test results, underlying Figure 2 in the main text

| Country | Type of Game | Low-high difference (percentage points) | t value | p value (two tailed) |
| --- | --- | --- | --- | --- |
| Pooled (cluster-adjusted) | MDG | 8.61 | 4.88 | p<0.05 |
| Germany | MDG | 7.43 | 4.90 | p<0.0001 |
| Poland | MDG | 4.75 | 2.56 | p<0.05 |
| Sweden | MDG | 13.61 | 8.22 | p<0.0001 |
| USA | MDG | 9.52 | 5.10 | p<0.0001 |
| Pooled (cluster-adjusted) | TDG | 2.46 | 1.88 | p>0.10 |
| Germany | TDG | 6.06 | 4.27 | p<0.0001 |
| Poland | TDG | 1.74 | 0.88 | p>0.10 |
| Sweden | TDG | 0.15 | 0.11 | p>0.10 |
| USA | TDG | 1.75 | 0.99 | p>0.10 |

**Table S4.** Additional robustness analyses for the pooled model; Cragg Hurdle Models on the percentage of money shared in the MDG

|  | M1 | M2 | M3 | M4 | M5 | M6 | M7 | M8 | M9 | M10 |
| --- | --- | --- | --- | --- | --- | --- | --- | --- | --- | --- |
| Percentage given MDG |  |  |  |  |  |  |  |  |  |  |
| Objective social status (composite, cent.) | 2.675*** (5.27) |  |  |  |  |  |  |  |  |  |
|  |  |  |  |  |  |  |  |  |  |  |
| Recipient: No status info | -7.033*** (-5.36) | -6.834*** (-4.78) | -6.655*** (-4.57) | -7.205*** (-3.59) | -8.929*** (-3.50) | -6.625*** (-4.77) | -7.162*** (-3.47) | -7.128*** (-3.48) | -7.217*** (-3.54) | -6.709*** (-4.59) |
|  |  |  |  |  |  |  |  |  |  |  |
| Recipient: Middle status | -5.345*** (-8.85) | -5.235*** (-7.75) | -5.202*** (-7.42) | -4.815*** (-6.35) | -6.656** (-2.67) | -5.080*** (-8.95) | -4.734*** (-6.05) | -4.748*** (-5.92) | -4.779*** (-6.36) | -5.171*** (-7.52) |
|  |  |  |  |  |  |  |  |  |  |  |
| Recipient: High status | -9.355*** (-6.61) | -9.253*** (-5.65) | -9.100*** (-5.57) | -7.670*** (-6.82) | -9.344*** (-5.13) | -9.045*** (-5.51) | -7.668*** (-6.59) | -7.618*** (-6.43) | -7.711*** (-6.89) | -9.155*** (-5.65) |
|  |  |  |  |  |  |  |  |  |  |  |
| Age | 0.0103 (0.20) | 0.0191 (0.38) | 0.00797 (0.16) | -0.00484 (-0.08) | -0.0110 (-0.21) | 0.00993 (0.20) | -0.0103 (-0.17) | -0.0134 (-0.22) | -0.00417 (-0.07) | 0.00984 (0.20) |
|  |  |  |  |  |  |  |  |  |  |  |
| Male | 2.596+ (1.78) | 3.148* (2.46) | 2.788* (2.21) | 3.726* (2.16) | 4.213+ (1.77) | 2.814* (2.28) | 3.670* (2.18) | 3.549* (2.12) | 3.804* (2.22) | 2.893* (2.23) |
|  |  |  |  |  |  |  |  |  |  |  |
| Education |  | 1.807*** (7.36) |  |  |  |  |  |  |  |  |
|  |  |  |  |  |  |  |  |  |  |  |
| Income deciles |  |  | 0.607*** (5.75) |  |  |  |  |  |  |  |
|  |  |  |  |  |  |  |  |  |  |  |
| Job prestige |  |  |  | 0.135* (2.41) |  |  |  |  |  |  |
|  |  |  |  |  |  |  |  |  |  |  |
| Job prestige (no imputations) |  |  |  |  | 0.147*** (3.73) |  |  |  |  |  |
|  |  |  |  |  |  |  |  |  |  |  |
| Income deciles (no imputations) |  |  |  |  |  | 0.634*** (5.50) |  |  |  |  |
|  |  |  |  |  |  |  |  |  |  |  |
| Social status (composite: education, income and job prestige) |  |  |  |  |  |  | 2.370*** (3.48) |  |  |  |
|  |  |  |  |  |  |  |  |  |  |  |
| Social status (composite: income and job prestige) |  |  |  |  |  |  |  | 2.326** (2.92) |  |  |
|  |  |  |  |  |  |  |  |  |  |  |
| Social status (composite: education and job prestige) |  |  |  |  |  |  |  |  | 2.088*** (3.54) |  |
|  |  |  |  |  |  |  |  |  |  |  |
| Social status (composite: education and income) |  |  |  |  |  |  |  |  |  | 2.286*** (6.45) |
|  |  |  |  |  |  |  |  |  |  |  |
| Constant | 47.13*** (9.53) | 39.80*** (8.52) | 43.60*** (8.36) | 38.54*** (5.42) | 38.77*** (4.63) | 43.30*** (8.52) | 44.54*** (6.54) | 44.73*** (6.46) | 44.23*** (6.49) | 46.79*** (8.76) |
| selection_ll |  |  |  |  |  |  |  |  |  |  |
| Objective social status (composite, cent.) | 0.0162 (1.02) |  |  |  |  |  |  |  |  |  |
|  |  |  |  |  |  |  |  |  |  |  |
| Recipient: No status info | -0.337*** (-7.65) | -0.303*** (-9.52) | -0.302*** (-9.29) | -0.315*** (-8.26) | -0.326*** (-6.52) | -0.305*** (-9.50) | -0.316*** (-8.23) | -0.316*** (-8.17) | -0.316*** (-8.34) | -0.303*** (-9.34) |
|  |  |  |  |  |  |  |  |  |  |  |
| Recipient: Middle status | -0.132+ (-1.90) | -0.112* (-1.96) | -0.112* (-1.97) | -0.0657* (-2.37) | -0.0466+ (-1.69) | -0.123* (-2.27) | -0.0655* (-2.39) | -0.0656* (-2.40) | -0.0656* (-2.36) | -0.112* (-1.96) |
|  |  |  |  |  |  |  |  |  |  |  |
| Recipient: High status | -0.249*** (-3.43) | -0.242*** (-4.43) | -0.241*** (-4.42) | -0.215*** (-4.06) | -0.164*** (-12.34) | -0.252*** (-4.58) | -0.215*** (-4.08) | -0.214*** (-4.07) | -0.215*** (-4.11) | -0.242*** (-4.41) |
|  |  |  |  |  |  |  |  |  |  |  |
| Age | 0.00917*** (9.37) | 0.00899*** (8.59) | 0.00888*** (8.43) | 0.00829*** (8.44) | 0.00641*** (3.71) | 0.00894*** (6.64) | 0.00824*** (8.32) | 0.00823*** (8.21) | 0.00827*** (8.35) | 0.00892*** (8.76) |
|  |  |  |  |  |  |  |  |  |  |  |
| Male | -0.0981* (-2.27) | -0.0850* (-2.04) | -0.0880* (-2.05) | -0.0734 (-1.29) | -0.0597* (-2.01) | -0.0896* (-2.22) | -0.0736 (-1.32) | -0.0742 (-1.32) | -0.0732 (-1.28) | -0.0864* (-2.10) |
|  |  |  |  |  |  |  |  |  |  |  |
| Education |  | 0.00238 (0.18) |  |  |  |  |  |  |  |  |
|  |  |  |  |  |  |  |  |  |  |  |
| Income deciles |  |  | 0.00607 (1.30) |  |  |  |  |  |  |  |
|  |  |  |  |  |  |  |  |  |  |  |
| Job prestige |  |  |  | 0.0000493 (0.03) |  |  |  |  |  |  |
|  |  |  |  |  |  |  |  |  |  |  |
| Job prestige (no imputations) |  |  |  |  | 0.00322*** (5.37) |  |  |  |  |  |
|  |  |  |  |  |  |  |  |  |  |  |
| Income deciles (no imputations) |  |  |  |  |  | 0.00571 (0.96) |  |  |  |  |
|  |  |  |  |  |  |  |  |  |  |  |
| Social status (composite: education, income and job prestige) |  |  |  |  |  |  | 0.00802 (0.53) |  |  |  |
|  |  |  |  |  |  |  |  |  |  |  |
| Social status (composite: income and job prestige) |  |  |  |  |  |  |  | 0.00842 (0.85) |  |  |
|  |  |  |  |  |  |  |  |  |  |  |
| Social status (composite: education and job prestige) |  |  |  |  |  |  |  |  | 0.00494 (0.21) |  |
|  |  |  |  |  |  |  |  |  |  |  |
| Social status (composite: education and income) |  |  |  |  |  |  |  |  |  | 0.0162 (1.37) |
|  |  |  |  |  |  |  |  |  |  |  |
| Constant | 0.990*** (4.93) | 0.968*** (4.59) | 0.949*** (5.52) | 0.935*** (3.46) | 0.881*** (3.63) | 0.955*** (5.20) | 0.940*** (4.16) | 0.940*** (4.14) | 0.938*** (4.16) | 0.980*** (5.13) |
| lnsigma |  |  |  |  |  |  |  |  |  |  |
| Constant | 3.253*** (36.93) | 3.266*** (36.15) | 3.267*** (36.55) | 3.312*** (29.74) | 3.247*** (31.03) | 3.264*** (36.58) | 3.311*** (29.57) | 3.311*** (29.69) | 3.312*** (29.58) | 3.265*** (36.28) |
| Observations | 7336 | 7722 | 7722 | 5811 | 2046 | 7575 | 5811 | 5811 | 5811 | 7722 |
| Log likelihood | -31916.5 | -33636.7 | -33641.4 | -25185.9 | -8797.9 | -32987.7 | -25180.6 | -25181.2 | -25183.8 | -33632.7 |

Note: z statistics in parentheses; + p<0.10, * p<0.05, ** p<0.01, *** p<0.001.

**Table S5.** Additional robustness analyses for Germany; Cragg Hurdle Models on the percentage of money shared in the MDG

|  | M1 | M2 | M3 | M4 | M5 | M6 | M7 | M8 | M9 | M10 |
| --- | --- | --- | --- | --- | --- | --- | --- | --- | --- | --- |
| Percentage given MDG |  |  |  |  |  |  |  |  |  |  |
| Objective social status (composite, cent.) | 2.053** (2.87) |  |  |  |  |  |  |  |  |  |
|  |  |  |  |  |  |  |  |  |  |  |
| Recipient: No status info | -5.787*** (-3.58) | -5.113** (-3.27) | -5.130** (-3.28) | -5.259*** (-3.36) | -5.619* (-2.27) | -5.260*** (-3.36) | -5.180*** (-3.31) | -5.221*** (-3.34) | -5.222*** (-3.34) | -5.080** (-3.25) |
|  |  |  |  |  |  |  |  |  |  |  |
| Recipient: Middle status | -4.127** (-2.63) | -4.068** (-2.68) | -4.017** (-2.64) | -4.159** (-2.73) | -2.534 (-1.01) | -4.116** (-2.70) | -4.036** (-2.65) | -4.067** (-2.67) | -4.111** (-2.70) | -3.975** (-2.61) |
|  |  |  |  |  |  |  |  |  |  |  |
| Recipient: High status | -6.793*** (-4.22) | -7.078*** (-4.53) | -6.965*** (-4.45) | -7.067*** (-4.51) | -8.418*** (-3.35) | -6.865*** (-4.37) | -7.020*** (-4.49) | -7.007*** (-4.48) | -7.075*** (-4.52) | -6.989*** (-4.47) |
|  |  |  |  |  |  |  |  |  |  |  |
| Age | 0.0163 (0.48) | 0.0144 (0.43) | -0.00955 (-0.29) | -0.00227 (-0.07) | -0.0181 (-0.32) | -0.0104 (-0.31) | 0.00382 (0.12) | -0.00454 (-0.14) | 0.00746 (0.22) | 0.00423 (0.13) |
|  |  |  |  |  |  |  |  |  |  |  |
| Male | 0.271 (0.24) | 1.027 (0.92) | 0.698 (0.63) | 0.891 (0.80) | 0.724 (0.40) | 0.916 (0.82) | 0.902 (0.81) | 0.816 (0.73) | 0.991 (0.89) | 0.850 (0.77) |
|  |  |  |  |  |  |  |  |  |  |  |
| Education |  | 1.431** (2.66) |  |  |  |  |  |  |  |  |
|  |  |  |  |  |  |  |  |  |  |  |
| Income deciles |  |  | 0.428* (2.17) |  |  |  |  |  |  |  |
|  |  |  |  |  |  |  |  |  |  |  |
| Job prestige |  |  |  | 0.0476 (1.20) |  |  |  |  |  |  |
|  |  |  |  |  |  |  |  |  |  |  |
| Job prestige (no imputations) |  |  |  |  | 0.131* (2.05) |  |  |  |  |  |
|  |  |  |  |  |  |  |  |  |  |  |
| Income deciles (no imputations) |  |  |  |  |  | 0.435* (2.19) |  |  |  |  |
|  |  |  |  |  |  |  |  |  |  |  |
| Social status (composite: education, income and job prestige) |  |  |  |  |  |  | 1.496** (2.65) |  |  |  |
|  |  |  |  |  |  |  |  |  |  |  |
| Social status (composite: income and job prestige) |  |  |  |  |  |  |  | 1.189* (2.12) |  |  |
|  |  |  |  |  |  |  |  |  |  |  |
| Social status (composite: education and job prestige) |  |  |  |  |  |  |  |  | 1.269* (2.23) |  |
|  |  |  |  |  |  |  |  |  |  |  |
| Social status (composite: education and income) |  |  |  |  |  |  |  |  |  | 1.668**  (2.95) |
|  |  |  |  |  |  |  |  |  |  |  |
| Constant | 46.87*** (22.29) | 41.39*** (13.49) | 45.35*** (20.12) | 45.31*** (16.59) | 42.37*** (9.27) | 45.44*** (20.15) | 46.96*** (23.14) | 47.43*** (23.47) | 46.78*** (22.86) | 46.91*** (23.13) |
| selection_ll |  |  |  |  |  |  |  |  |  |  |
| Objective social status (composite, cent.) | -0.00655 (-0.12) |  |  |  |  |  |  |  |  |  |
|  |  |  |  |  |  |  |  |  |  |  |
| Recipient: No status info | -0.393** (-3.15) | -0.349** (-3.00) | -0.349** (-3.00) | -0.350** (-3.01) | -0.298 (-1.50) | -0.393*** (-3.30) | -0.349** (-3.00) | -0.350** (-3.01) | -0.349** (-3.01) | -0.349** (-3.00) |
|  |  |  |  |  |  |  |  |  |  |  |
| Recipient: Middle status | -0.106 (-0.80) | -0.0716 (-0.58) | -0.0673 (-0.54) | -0.0703 (-0.57) | -0.101 (-0.47) | -0.125 (-0.99) | -0.0681 (-0.55) | -0.0670 (-0.54) | -0.0704 (-0.57) | -0.0684 (-0.55) |
|  |  |  |  |  |  |  |  |  |  |  |
| Recipient: High status | -0.255* (-1.99) | -0.254* (-2.14) | -0.252* (-2.12) | -0.253* (-2.13) | -0.172 (-0.83) | -0.301* (-2.47) | -0.252* (-2.11) | -0.251* (-2.11) | -0.253* (-2.13) | -0.252* (-2.12) |
|  |  |  |  |  |  |  |  |  |  |  |
| Age | 0.00680* (2.57) | 0.00696** (2.75) | 0.00658** (2.68) | 0.00679** (2.75) | 0.00249 (0.55) | 0.00615* (2.47) | 0.00689** (2.78) | 0.00673** (2.74) | 0.00693** (2.77) | 0.00687** (2.78) |
|  |  |  |  |  |  |  |  |  |  |  |
| Male | -0.183* (-2.07) | -0.140+ (-1.72) | -0.146+ (-1.78) | -0.141+ (-1.72) | -0.0913 (-0.63) | -0.159+ (-1.91) | -0.142+ (-1.73) | -0.143+ (-1.75) | -0.140+ (-1.71) | -0.143+ (-1.75) |
|  |  |  |  |  |  |  |  |  |  |  |
| Education |  | 0.0176 (0.46) |  |  |  |  |  |  |  |  |
|  |  |  |  |  |  |  |  |  |  |  |
| Income deciles |  |  | 0.00962 (0.68) |  |  |  |  |  |  |  |
|  |  |  |  |  |  |  |  |  |  |  |
| Job prestige |  |  |  | 0.00120 (0.41) |  |  |  |  |  |  |
|  |  |  |  |  |  |  |  |  |  |  |
| Job prestige (no imputations) |  |  |  |  | 0.00443 (0.84) |  |  |  |  |  |
|  |  |  |  |  |  |  |  |  |  |  |
| Income deciles (no imputations) |  |  |  |  |  | 0.00420 (0.29) |  |  |  |  |
|  |  |  |  |  |  |  |  |  |  |  |
| Social status (composite: education, income and job prestige) |  |  |  |  |  |  | 0.0273 (0.68) |  |  |  |
|  |  |  |  |  |  |  |  |  |  |  |
| Social status (composite: income and job prestige) |  |  |  |  |  |  |  | 0.0272 (0.68) |  |  |
|  |  |  |  |  |  |  |  |  |  |  |
| Social status (composite: education and job prestige) |  |  |  |  |  |  |  |  | 0.0207 (0.51) |  |
|  |  |  |  |  |  |  |  |  |  |  |
| Social status (composite: education and income) |  |  |  |  |  |  |  |  |  | 0.0281  (0.70) |
|  |  |  |  |  |  |  |  |  |  |  |
| Constant | 1.362*** (8.28) | 1.221*** (5.47) | 1.249*** (7.50) | 1.239*** (6.00) | 1.307*** (3.47) | 1.352*** (7.90) | 1.284*** (8.42) | 1.293*** (8.53) | 1.282*** (8.32) | 1.286*** (8.45) |
| lnsigma |  |  |  |  |  |  |  |  |  |  |
| Constant | 3.080*** (144.16) | 3.092*** (151.00) | 3.093*** (150.98) | 3.094*** (150.96) | 3.051*** (88.95) | 3.090*** (150.22) | 3.092*** (151.00) | 3.093*** (150.98) | 3.093*** (150.99) | 3.092*** (151.02) |
| Observations | 1766 | 1962 | 1962 | 1962 | 666 | 1930 | 1962 | 1962 | 1962 | 1962 |
| Log likelihood | -7657.7 | -8505.0 | -8506.1 | -8507.9 | -2880.3 | -8370.0 | -8504.9 | -8506.2 | -8506.1 | -8504.1 |

Note: z statistics in parentheses; + p<0.10, * p<0.05, ** p<0.01, *** p<0.001.

**Table S6.** Additional robustness analyses for Poland; Cragg Hurdle Models on the percentage of money shared in the MDG

|  | M1 | M2 | M3 | M4 | M5 | M6 | M7 | M8 | M9 | M10 |
| --- | --- | --- | --- | --- | --- | --- | --- | --- | --- | --- |
| Percentage given MDG |  |  |  |  |  |  |  |  |  |  |
| Objective social status (composite, cent.) | 0.736 (0.54) |  |  |  |  |  |  |  |  |  |
|  |  |  |  |  |  |  |  |  |  |  |
| Recipient: No status info | -5.606+ (-1.85) | -5.327+ (-1.81) | -5.133+ (-1.74) | -5.339+ (-1.82) | -8.072* (-2.09) | -5.221+ (-1.77) | -5.271+ (-1.79) | -5.157+ (-1.75) | -5.388+ (-1.83) | -5.243+ (-1.78) |
|  |  |  |  |  |  |  |  |  |  |  |
| Recipient: Middle status | -3.564 (-1.21) | -4.326 (-1.49) | -4.272 (-1.47) | -4.266 (-1.47) | -8.858* (-2.22) | -4.464 (-1.54) | -4.248 (-1.47) | -4.214 (-1.46) | -4.286 (-1.48) | -4.285 (-1.48) |
|  |  |  |  |  |  |  |  |  |  |  |
| Recipient: High status | -5.166+ (-1.72) | -6.050* (-2.05) | -5.883* (-1.99) | -5.993* (-2.04) | -7.170+ (-1.82) | -5.887* (-2.00) | -5.994* (-2.03) | -5.872* (-1.99) | -6.083* (-2.06) | -5.987* (-2.03) |
|  |  |  |  |  |  |  |  |  |  |  |
| Age | 0.0364 (0.48) | 0.0963 (1.33) | 0.0923 (1.28) | 0.0711 (0.98) | 0.0484 (0.48) | 0.0858 (1.19) | 0.0683 (0.92) | 0.0649 (0.88) | 0.0739 (1.01) | 0.0865 (1.18) |
|  |  |  |  |  |  |  |  |  |  |  |
| Male | 5.980** (2.75) | 7.132*** (3.35) | 6.935** (3.23) | 6.890** (3.24) | 8.688** (3.02) | 6.600** (3.08) | 6.744** (3.15) | 6.636** (3.10) | 6.916** (3.25) | 6.936** (3.24) |
|  |  |  |  |  |  |  |  |  |  |  |
| Education |  | 0.757 (0.72) |  |  |  |  |  |  |  |  |
|  |  |  |  |  |  |  |  |  |  |  |
| Income deciles |  |  | 0.403 (1.06) |  |  |  |  |  |  |  |
|  |  |  |  |  |  |  |  |  |  |  |
| Job prestige |  |  |  | 0.154* (1.97) |  |  |  |  |  |  |
|  |  |  |  |  |  |  |  |  |  |  |
| Job prestige (no imputations) |  |  |  |  | 0.200+ (1.93) |  |  |  |  |  |
|  |  |  |  |  |  |  |  |  |  |  |
| Income deciles (no imputations) |  |  |  |  |  | 0.418 (1.09) |  |  |  |  |
|  |  |  |  |  |  |  |  |  |  |  |
| Social status (composite: education, income and job prestige) |  |  |  |  |  |  | 1.906+ (1.71) |  |  |  |
|  |  |  |  |  |  |  |  |  |  |  |
| Social status (composite: income and job prestige) |  |  |  |  |  |  |  | 2.186* (1.96) |  |  |
|  |  |  |  |  |  |  |  |  |  |  |
| Social status (composite: education and job prestige) |  |  |  |  |  |  |  |  | 1.760 (1.60) |  |
|  |  |  |  |  |  |  |  |  |  |  |
| Social status (composite: education and income) |  |  |  |  |  |  |  |  |  | 1.207  (1.10) |
|  |  |  |  |  |  |  |  |  |  |  |
| Constant | 31.15*** (7.69) | 25.18*** (5.09) | 25.92*** (6.26) | 22.50*** (4.83) | 24.68*** (3.95) | 26.35*** (6.39) | 29.27*** (7.30) | 29.41*** (7.38) | 29.00*** (7.26) | 28.41*** (7.12) |
| selection_ll |  |  |  |  |  |  |  |  |  |  |
| Objective social status (composite, cent.) | -0.00561 (-0.13) |  |  |  |  |  |  |  |  |  |
|  |  |  |  |  |  |  |  |  |  |  |
| Recipient: No status info | -0.273** (-2.84) | -0.269** (-2.93) | -0.268** (-2.92) | -0.268** (-2.93) | -0.379** (-2.76) | -0.271** (-2.93) | -0.268** (-2.93) | -0.268** (-2.92) | -0.269** (-2.94) | -0.268** (-2.92) |
|  |  |  |  |  |  |  |  |  |  |  |
| Recipient: Middle status | -0.0403 (-0.40) | -0.0387 (-0.40) | -0.0387 (-0.41) | -0.0393 (-0.41) | -0.0252 (-0.17) | -0.0416 (-0.43) | -0.0393 (-0.41) | -0.0394 (-0.41) | -0.0389 (-0.41) | -0.0391 (-0.41) |
|  |  |  |  |  |  |  |  |  |  |  |
| Recipient: High status | -0.137 (-1.39) | -0.145 (-1.55) | -0.144 (-1.54) | -0.143 (-1.53) | -0.157 (-1.07) | -0.150 (-1.59) | -0.144 (-1.53) | -0.143 (-1.52) | -0.145 (-1.55) | -0.144 (-1.53) |
|  |  |  |  |  |  |  |  |  |  |  |
| Age | 0.00721** (2.94) | 0.00608** (2.66) | 0.00644** (2.82) | 0.00586* (2.53) | 0.00606+ (1.67) | 0.00715** (3.10) | 0.00596* (2.54) | 0.00606** (2.59) | 0.00582* (2.50) | 0.00622** (2.69) |
|  |  |  |  |  |  |  |  |  |  |  |
| Male | -0.00622 (-0.09) | 0.00911 (0.14) | 0.0126 (0.19) | 0.00719 (0.11) | -0.0151 (-0.15) | 0.00306 (0.05) | 0.00641 (0.10) | 0.00687 (0.10) | 0.00719 (0.11) | 0.00890 (0.13) |
|  |  |  |  |  |  |  |  |  |  |  |
| Education |  | 0.0188 (0.57) |  |  |  |  |  |  |  |  |
|  |  |  |  |  |  |  |  |  |  |  |
| Income deciles |  |  | -0.00241 (-0.20) |  |  |  |  |  |  |  |
|  |  |  |  |  |  |  |  |  |  |  |
| Job prestige |  |  |  | 0.00209 (0.85) |  |  |  |  |  |  |
|  |  |  |  |  |  |  |  |  |  |  |
| Job prestige (no imputations) |  |  |  |  | 0.00297 (0.80) |  |  |  |  |  |
|  |  |  |  |  |  |  |  |  |  |  |
| Income deciles (no imputations) |  |  |  |  |  | -0.00487 (-0.41) |  |  |  |  |
|  |  |  |  |  |  |  |  |  |  |  |
| Social status (composite: education, income and job prestige) |  |  |  |  |  |  | 0.0196 (0.56) |  |  |  |
|  |  |  |  |  |  |  |  |  |  |  |
| Social status (composite: income and job prestige) |  |  |  |  |  |  |  | 0.0147 (0.42) |  |  |
|  |  |  |  |  |  |  |  |  |  |  |
| Social status (composite: education and job prestige) |  |  |  |  |  |  |  |  | 0.0294 (0.85) |  |
|  |  |  |  |  |  |  |  |  |  |  |
| Social status (composite: education and income) |  |  |  |  |  |  |  |  |  | 0.00779  (0.23) |
|  |  |  |  |  |  |  |  |  |  |  |
| Constant | 0.641*** (5.13) | 0.628*** (4.14) | 0.692*** (5.66) | 0.617*** (4.43) | 0.646** (2.98) | 0.679*** (5.53) | 0.702*** (5.86) | 0.698*** (5.82) | 0.709*** (5.96) | 0.690*** (5.82) |
| lnsigma |  |  |  |  |  |  |  |  |  |  |
| Constant | 3.456*** (104.45) | 3.480*** (109.44) | 3.480*** (109.46) | 3.479*** (109.57) | 3.394*** (75.60) | 3.475*** (108.73) | 3.479*** (109.53) | 3.479*** (109.57) | 3.479*** (109.52) | 3.480*** (109.47) |
| Observations | 1733 | 1924 | 1924 | 1924 | 826 | 1899 | 1924 | 1924 | 1924 | 1924 |
| Log likelihood | -7147.9 | -7981.5 | -7981.3 | -7979.6 | -3414.8 | -7864.8 | -7980.3 | -7979.9 | -7980.2 | -7981.2 |

Note: z statistics in parentheses; + p<0.10, * p<0.05, ** p<0.01, *** p<0.001.

**Table S7.** Additional robustness analyses for Sweden; Cragg Hurdle Models on the percentage of money shared in the MDG

|  | M1 | M2 | M3 | M4 | M10 |
| --- | --- | --- | --- | --- | --- |
| Percentage given MDG |  |  |  |  |  |
| Objective social status (composite, cent.) | 3.114*** (4.41) |  |  |  |  |
|  |  |  |  |  |  |
| Recipient: No status info | -5.737*** (-3.46) | -6.224*** (-3.93) | -5.871*** (-3.69) | -5.758*** (-3.57) | -5.898*** (-3.73) |
|  |  |  |  |  |  |
| Recipient: Middle status | -5.706*** (-3.48) | -6.549*** (-4.19) | -6.409*** (-4.09) | -6.145*** (-3.86) | -6.412*** (-4.11) |
|  |  |  |  |  |  |
| Recipient: High status | -12.63*** (-7.64) | -13.29*** (-8.34) | -13.03*** (-8.14) | -13.04*** (-8.06) | -13.07*** (-8.20) |
|  |  |  |  |  |  |
| Age | 0.0482 (1.33) | 0.0369 (1.09) | 0.0174 (0.51) | 0.0218 (0.63) | 0.0273 (0.81) |
|  |  |  |  |  |  |
| Male | 0.149 (0.11) | 1.231 (1.01) | 1.001 (0.81) | 0.793 (0.63) | 0.763 (0.62) |
|  |  |  |  |  |  |
| Education |  | 2.196*** (4.71) |  |  |  |
|  |  |  |  |  |  |
| Income deciles |  |  | 0.632** (3.10) |  |  |
|  |  |  |  |  |  |
| Income deciles (no imputations) |  |  |  | 0.690*** (3.33) |  |
|  |  |  |  |  |  |
| Social status (composite: education and income) |  |  |  |  | 2.878***  (4.97) |
|  |  |  |  |  |  |
| Constant | 52.98*** (26.81) | 45.47*** (17.36) | 50.91*** (23.82) | 50.46*** (23.35) | 53.99*** (28.90) |
| selection_ll |  |  |  |  |  |
| Objective social status (composite, cent.) | 0.0570 (1.10) |  |  |  |  |
|  |  |  |  |  |  |
| Recipient: No status info | -0.282* (-2.11) | -0.240+ (-1.89) | -0.232+ (-1.83) | -0.237+ (-1.86) | -0.236+ (-1.86) |
|  |  |  |  |  |  |
| Recipient: Middle status | -0.354** (-2.70) | -0.306* (-2.48) | -0.302* (-2.45) | -0.289* (-2.31) | -0.305* (-2.47) |
|  |  |  |  |  |  |
| Recipient: High status | -0.412** (-3.20) | -0.370** (-3.03) | -0.368** (-3.02) | -0.358** (-2.91) | -0.370** (-3.03) |
|  |  |  |  |  |  |
| Age | 0.00967*** (3.55) | 0.0102*** (3.99) | 0.00987*** (3.84) | 0.0103*** (3.92) | 0.0101*** (3.95) |
|  |  |  |  |  |  |
| Male | -0.166+ (-1.73) | -0.131 (-1.44) | -0.140 (-1.53) | -0.137 (-1.48) | -0.140 (-1.53) |
|  |  |  |  |  |  |
| Education |  | 0.0237 (0.69) |  |  |  |
|  |  |  |  |  |  |
| Income deciles |  |  | 0.0176 (1.20) |  |  |
|  |  |  |  |  |  |
| Income deciles (no imputations) |  |  |  | 0.0186 (1.26) |  |
|  |  |  |  |  |  |
| Social status (composite: education and income) |  |  |  |  | 0.0522  (1.22) |
|  |  |  |  |  |  |
| Constant | 1.255*** (8.40) | 1.100*** (5.78) | 1.110*** (7.20) | 1.073*** (6.93) | 1.198*** (8.52) |
| lnsigma |  |  |  |  |  |
| Constant | 3.110*** (150.77) | 3.114*** (157.84) | 3.118*** (157.79) | 3.115*** (155.45) | 3.113*** (157.84) |
| Observations | 1743 | 1911 | 1911 | 1853 | 1911 |
| Log likelihood | -7614.6 | -8361.3 | -8367.1 | -8104.6 | -8359.5 |

Note: z statistics in parentheses; + p<0.10, * p<0.05, ** p<0.01, *** p<0.001.

**Table S8.** Additional robustness analyses for USA; Cragg Hurdle Models on the percentage of money shared in the MDG

|  | M1 | M2 | M3 | M4 | M5 | M6 | M7 | M8 | M9 | M10 |
| --- | --- | --- | --- | --- | --- | --- | --- | --- | --- | --- |
| Percentage given MDG |  |  |  |  |  |  |  |  |  |  |
| Objective social status (composite, cent.) | 3.953*** (3.95) |  |  |  |  |  |  |  |  |  |
|  |  |  |  |  |  |  |  |  |  |  |
| Recipient: No status info | -11.87*** (-5.12) | -11.22*** (-5.05) | -11.23*** (-5.06) | -11.01*** (-4.97) | -15.45*** (-4.33) | -10.99*** (-4.93) | -11.15*** (-5.05) | -11.10*** (-5.03) | -11.11*** (-5.02) | -11.25*** (-5.08) |
|  |  |  |  |  |  |  |  |  |  |  |
| Recipient: Middle status | -5.727* (-2.56) | -6.349** (-2.94) | -6.557** (-3.04) | -6.317** (-2.94) | -11.39** (-3.13) | -6.171** (-2.84) | -6.323** (-2.95) | -6.411** (-2.99) | -6.263** (-2.91) | -6.410** (-2.98) |
|  |  |  |  |  |  |  |  |  |  |  |
| Recipient: High status | -10.60*** (-4.61) | -9.899*** (-4.48) | -10.05*** (-4.55) | -9.840*** (-4.46) | -14.39*** (-3.90) | -10.05*** (-4.51) | -9.984*** (-4.54) | -10.01*** (-4.55) | -9.872*** (-4.48) | -10.03*** (-4.55) |
|  |  |  |  |  |  |  |  |  |  |  |
| Age | -0.108* (-2.34) | -0.0954* (-2.14) | -0.0953* (-2.14) | -0.0987* (-2.22) | -0.109 (-1.46) | -0.0907* (-2.02) | -0.110* (-2.47) | -0.108* (-2.42) | -0.104* (-2.33) | -0.104* (-2.34) |
|  |  |  |  |  |  |  |  |  |  |  |
| Male | 5.569*** (3.38) | 4.839** (3.05) | 4.331** (2.75) | 4.651** (2.95) | 3.954 (1.52) | 4.415** (2.79) | 4.928** (3.13) | 4.629** (2.95) | 4.957** (3.14) | 4.803** (3.04) |
|  |  |  |  |  |  |  |  |  |  |  |
| Education |  | 2.005** (3.03) |  |  |  |  |  |  |  |  |
|  |  |  |  |  |  |  |  |  |  |  |
| Income deciles |  |  | 0.927*** (3.38) |  |  |  |  |  |  |  |
|  |  |  |  |  |  |  |  |  |  |  |
| Job prestige |  |  |  | 0.227*** (4.01) |  |  |  |  |  |  |
|  |  |  |  |  |  |  |  |  |  |  |
| Job prestige (no imputations) |  |  |  |  | 0.0616 (0.65) |  |  |  |  |  |
|  |  |  |  |  |  |  |  |  |  |  |
| Income deciles (no imputations) |  |  |  |  |  | 0.958*** (3.47) |  |  |  |  |
|  |  |  |  |  |  |  |  |  |  |  |
| Social status (composite: education, income and job prestige) |  |  |  |  |  |  | 3.740*** (4.70) |  |  |  |
|  |  |  |  |  |  |  |  |  |  |  |
| Social status (composite: income and job prestige) |  |  |  |  |  |  |  | 3.720*** (4.72) |  |  |
|  |  |  |  |  |  |  |  |  |  |  |
| Social status (composite: education and job prestige) |  |  |  |  |  |  |  |  | 3.276*** (4.11) |  |
|  |  |  |  |  |  |  |  |  |  |  |
| Social status (composite: education and income) |  |  |  |  |  |  |  |  |  | 3.233***  (4.02) |
|  |  |  |  |  |  |  |  |  |  |  |
| Constant | 53.56*** (19.53) | 45.86*** (13.07) | 48.46*** (16.48) | 43.72*** (12.51) | 54.50*** (8.96) | 47.74*** (16.08) | 53.88*** (20.56) | 53.93*** (20.57) | 53.51*** (20.39) | 53.68*** (20.43) |
| selection_ll |  |  |  |  |  |  |  |  |  |  |
| Objective social status (composite, cent.) | -0.00593 (-0.12) |  |  |  |  |  |  |  |  |  |
|  |  |  |  |  |  |  |  |  |  |  |
| Recipient: No status info | -0.453*** (-3.81) | -0.383*** (-3.45) | -0.387*** (-3.49) | -0.385*** (-3.47) | -0.228 (-1.08) | -0.360** (-3.20) | -0.384*** (-3.47) | -0.385*** (-3.48) | -0.383*** (-3.46) | -0.385*** (-3.48) |
|  |  |  |  |  |  |  |  |  |  |  |
| Recipient: Middle status | -0.140 (-1.11) | -0.129 (-1.10) | -0.130 (-1.11) | -0.128 (-1.10) | -0.0802 (-0.36) | -0.144 (-1.23) | -0.127 (-1.09) | -0.127 (-1.09) | -0.129 (-1.10) | -0.127 (-1.09) |
|  |  |  |  |  |  |  |  |  |  |  |
| Recipient: High status | -0.340** (-2.81) | -0.296** (-2.62) | -0.301** (-2.66) | -0.299** (-2.64) | -0.176 (-0.80) | -0.308** (-2.70) | -0.298** (-2.63) | -0.299** (-2.65) | -0.296** (-2.62) | -0.299** (-2.64) |
|  |  |  |  |  |  |  |  |  |  |  |
| Age | 0.00548* (2.36) | 0.00661** (2.98) | 0.00626** (2.83) | 0.00655** (2.96) | 0.00240 (0.56) | 0.00567* (2.53) | 0.00653** (2.94) | 0.00642** (2.90) | 0.00667** (3.01) | 0.00641** (2.89) |
|  |  |  |  |  |  |  |  |  |  |  |
| Male | -0.125 (-1.54) | -0.159* (-2.06) | -0.149+ (-1.94) | -0.151* (-1.97) | -0.113 (-0.76) | -0.141+ (-1.82) | -0.153* (-2.00) | -0.150+ (-1.96) | -0.158* (-2.05) | -0.151* (-1.97) |
|  |  |  |  |  |  |  |  |  |  |  |
| Education |  | -0.0316 (-0.99) |  |  |  |  |  |  |  |  |
|  |  |  |  |  |  |  |  |  |  |  |
| Income deciles |  |  | 0.00801 (0.61) |  |  |  |  |  |  |  |
|  |  |  |  |  |  |  |  |  |  |  |
| Job prestige |  |  |  | -0.00309 (-1.12) |  |  |  |  |  |  |
|  |  |  |  |  |  |  |  |  |  |  |
| Job prestige (no imputations) |  |  |  |  | 0.00165 (0.31) |  |  |  |  |  |
|  |  |  |  |  |  |  |  |  |  |  |
| Income deciles (no imputations) |  |  |  |  |  | 0.0130 (0.98) |  |  |  |  |
|  |  |  |  |  |  |  |  |  |  |  |
| Social status (composite: education, income and job prestige) |  |  |  |  |  |  | -0.0259 (-0.67) |  |  |  |
|  |  |  |  |  |  |  |  |  |  |  |
| Social status (composite: income and job prestige) |  |  |  |  |  |  |  | -0.0122 (-0.32) |  |  |
|  |  |  |  |  |  |  |  |  |  |  |
| Social status (composite: education and job prestige) |  |  |  |  |  |  |  |  | -0.0482 (-1.24) |  |
|  |  |  |  |  |  |  |  |  |  |  |
| Social status (composite: education and income) |  |  |  |  |  |  |  |  |  | -0.00887  (-0.23) |
|  |  |  |  |  |  |  |  |  |  |  |
| Constant | 1.301*** (9.08) | 1.337*** (7.77) | 1.191*** (8.10) | 1.353*** (7.80) | 1.308*** (3.63) | 1.191*** (8.02) | 1.222*** (9.17) | 1.226*** (9.20) | 1.218*** (9.15) | 1.227*** (9.21) |
| lnsigma |  |  |  |  |  |  |  |  |  |  |
| Constant | 3.346*** (133.41) | 3.357*** (139.97) | 3.356*** (140.02) | 3.354*** (140.12) | 3.254*** (76.88) | 3.354*** (138.82) | 3.352*** (140.24) | 3.352*** (140.25) | 3.354*** (140.13) | 3.354*** (140.12) |
| Observations | 1734 | 1925 | 1925 | 1925 | 554 | 1893 | 1925 | 1925 | 1925 | 1925 |
| Log likelihood | -7688.7 | -8540.3 | -8539.5 | -8536.8 | -2443.0 | -8403.8 | -8534.1 | -8534.2 | -8536.2 | -8537.3 |

Note: z statistics in parentheses; + p<0.10, * p<0.05, ** p<0.01, *** p<0.001.

**Table S9.** Additional robustness analyses for the pooled model; Cragg Hurdle Models on the percentage of time kept in the TDG

|  | M1 | M2 | M3 | M4 | M5 | M6 | M7 | M8 | M9 | M10 |
| --- | --- | --- | --- | --- | --- | --- | --- | --- | --- | --- |
| Percentage kept TDG |  |  |  |  |  |  |  |  |  |  |
| Objective social status (composite, cent.) | -1.958*** (-3.68) |  |  |  |  |  |  |  |  |  |
|  |  |  |  |  |  |  |  |  |  |  |
| Recipient: No status info | 1.197 (1.33) | 0.845 (1.10) | 0.741 (1.02) | 0.510 (0.61) | 0.563 (0.18) | 0.560 (0.73) | 0.514 (0.62) | 0.487 (0.59) | 0.542 (0.64) | 0.792 (1.08) |
|  |  |  |  |  |  |  |  |  |  |  |
| Recipient: Middle status | -0.987 (-0.78) | -1.262 (-1.06) | -1.286 (-1.10) | -2.337** (-3.20) | -2.699*** (-4.31) | -1.480 (-1.40) | -2.347** (-3.23) | -2.336** (-3.21) | -2.352** (-3.24) | -1.267 (-1.06) |
|  |  |  |  |  |  |  |  |  |  |  |
| Recipient: High status | -2.433 (-1.59) | -2.388 (-1.60) | -2.450+ (-1.66) | -3.578** (-2.95) | -1.124 (-0.61) | -2.635+ (-1.79) | -3.616** (-2.97) | -3.619** (-2.96) | -3.579** (-2.96) | -2.417 (-1.61) |
|  |  |  |  |  |  |  |  |  |  |  |
| Age | -0.108*** (-4.11) | -0.114*** (-4.24) | -0.108*** (-3.30) | -0.0979*** (-4.45) | -0.0530+ (-1.78) | -0.105** (-3.02) | -0.0936*** (-4.30) | -0.0943*** (-3.67) | -0.0966*** (-5.04) | -0.108*** (-3.80) |
|  |  |  |  |  |  |  |  |  |  |  |
| Male | -0.452 (-0.58) | -0.901 (-1.11) | -0.673 (-0.81) | -1.372+ (-1.65) | 0.795 (1.59) | -0.634 (-0.78) | -1.342+ (-1.70) | -1.268 (-1.50) | -1.464+ (-1.89) | -0.703 (-0.87) |
|  |  |  |  |  |  |  |  |  |  |  |
| Education |  | -1.486*** (-5.91) |  |  |  |  |  |  |  |  |
|  |  |  |  |  |  |  |  |  |  |  |
| Income deciles |  |  | -0.311 (-1.22) |  |  |  |  |  |  |  |
|  |  |  |  |  |  |  |  |  |  |  |
| Job prestige |  |  |  | -0.0845*** (-5.48) |  |  |  |  |  |  |
|  |  |  |  |  |  |  |  |  |  |  |
| Job prestige (no imputations) |  |  |  |  | -0.158*** (-7.32) |  |  |  |  |  |
|  |  |  |  |  |  |  |  |  |  |  |
| Income deciles (no imputations) |  |  |  |  |  | -0.348 (-1.26) |  |  |  |  |
|  |  |  |  |  |  |  |  |  |  |  |
| Social status (composite: education, income and job prestige) |  |  |  |  |  |  | -1.695* (-2.53) |  |  |  |
|  |  |  |  |  |  |  |  |  |  |  |
| Social status (composite: income and job prestige) |  |  |  |  |  |  |  | -1.256+ (-1.69) |  |  |
|  |  |  |  |  |  |  |  |  |  |  |
| Social status (composite: education and job prestige) |  |  |  |  |  |  |  |  | -1.788*** (-5.99) |  |
|  |  |  |  |  |  |  |  |  |  |  |
| Social status (composite: education and income) |  |  |  |  |  |  |  |  |  | -1.628** (-2.78) |
|  |  |  |  |  |  |  |  |  |  |  |
| Constant | 59.01*** (17.32) | 64.91*** (21.63) | 60.89*** (23.75) | 64.21*** (19.16) | 64.59*** (18.04) | 61.10*** (23.94) | 60.41*** (15.24) | 60.41*** (14.43) | 60.59*** (15.86) | 59.16*** (16.76) |
| selection_ll |  |  |  |  |  |  |  |  |  |  |
| Objective social status (composite, cent.) | -0.00449 (-0.11) |  |  |  |  |  |  |  |  |  |
|  |  |  |  |  |  |  |  |  |  |  |
| Recipient: No status info | -0.235** (-2.86) | -0.253** (-3.14) | -0.253** (-3.15) | -0.192*** (-3.58) | -0.220* (-2.49) | -0.254*** (-3.48) | -0.193*** (-3.57) | -0.193*** (-3.60) | -0.193*** (-3.53) | -0.253** (-3.15) |
|  |  |  |  |  |  |  |  |  |  |  |
| Recipient: Middle status | -0.118 (-1.05) | -0.108 (-0.93) | -0.108 (-0.92) | -0.0318 (-0.32) | -0.0130 (-0.09) | -0.0896 (-0.81) | -0.0311 (-0.31) | -0.0308 (-0.31) | -0.0315 (-0.32) | -0.108 (-0.92) |
|  |  |  |  |  |  |  |  |  |  |  |
| Recipient: High status | -0.0486 (-0.67) | -0.0577 (-0.85) | -0.0577 (-0.84) | -0.0197 (-0.32) | 0.00933 (0.08) | -0.0559 (-0.84) | -0.0205 (-0.33) | -0.0204 (-0.33) | -0.0205 (-0.33) | -0.0576 (-0.84) |
|  |  |  |  |  |  |  |  |  |  |  |
| Age | 0.00324*** (4.49) | 0.00328*** (3.92) | 0.00320*** (4.34) | 0.00267*** (5.83) | 0.00329+ (1.96) | 0.00276*** (3.64) | 0.00259*** (6.18) | 0.00267*** (8.21) | 0.00258*** (5.37) | 0.00323*** (3.95) |
|  |  |  |  |  |  |  |  |  |  |  |
| Male | -0.227*** (-5.92) | -0.210*** (-5.60) | -0.212*** (-5.57) | -0.199*** (-3.87) | -0.248*** (-3.55) | -0.199*** (-5.27) | -0.199*** (-3.95) | -0.197*** (-3.97) | -0.200*** (-3.99) | -0.210*** (-5.37) |
|  |  |  |  |  |  |  |  |  |  |  |
| Education |  | -0.00429 (-0.15) |  |  |  |  |  |  |  |  |
|  |  |  |  |  |  |  |  |  |  |  |
| Income deciles |  |  | 0.00388 (0.59) |  |  |  |  |  |  |  |
|  |  |  |  |  |  |  |  |  |  |  |
| Job prestige |  |  |  | -0.00289 (-0.86) |  |  |  |  |  |  |
|  |  |  |  |  |  |  |  |  |  |  |
| Job prestige (no imputations) |  |  |  |  | -0.00290 (-0.57) |  |  |  |  |  |
|  |  |  |  |  |  |  |  |  |  |  |
| Income deciles (no imputations) |  |  |  |  |  | 0.00312 (0.49) |  |  |  |  |
|  |  |  |  |  |  |  |  |  |  |  |
| Social status (composite: education, income and job prestige) |  |  |  |  |  |  | -0.0146 (-0.32) |  |  |  |
|  |  |  |  |  |  |  |  |  |  |  |
| Social status (composite: income and job prestige) |  |  |  |  |  |  |  | -0.0238 (-0.55) |  |  |
|  |  |  |  |  |  |  |  |  |  |  |
| Social status (composite: education and job prestige) |  |  |  |  |  |  |  |  | -0.0202 (-0.42) |  |
|  |  |  |  |  |  |  |  |  |  |  |
| Social status (composite: education and income) |  |  |  |  |  |  |  |  |  | 0.00870 (0.30) |
|  |  |  |  |  |  |  |  |  |  |  |
| Constant | 1.573*** (9.25) | 1.584*** (18.47) | 1.552*** (10.66) | 1.632*** (8.66) | 1.665*** (5.90) | 1.570*** (10.91) | 1.512*** (8.42) | 1.507*** (8.54) | 1.513*** (8.47) | 1.571*** (9.66) |
| lnsigma |  |  |  |  |  |  |  |  |  |  |
| Constant | 3.219*** (35.89) | 3.221*** (37.44) | 3.223*** (37.37) | 3.270*** (39.47) | 3.265*** (39.74) | 3.221*** (37.29) | 3.269*** (39.17) | 3.270*** (39.25) | 3.269*** (39.37) | 3.221*** (37.21) |
| Observations | 7336 | 7722 | 7722 | 5811 | 2046 | 7575 | 5811 | 5811 | 5811 | 7722 |
| Log likelihood | -33084.0 | -34845.1 | -34855.7 | -26386.3 | -9300.5 | -34197.1 | -26382.4 | -26386.6 | -26381.1 | -34846.3 |

Note: z statistics in parentheses; + p<0.10, * p<0.05, ** p<0.01, *** p<0.001.

**Table S10.** Additional robustness analyses for Germany; Cragg Hurdle Models on the percentage of time kept in the TDG

|  | M1 | M2 | M3 | M4 | M5 | M6 | M7 | M8 | M9 | M10 |
| --- | --- | --- | --- | --- | --- | --- | --- | --- | --- | --- |
| Percentage kept TDG |  |  |  |  |  |  |  |  |  |  |
| Objective social status (composite, cent.) | -1.947** (-2.85) |  |  |  |  |  |  |  |  |  |
|  |  |  |  |  |  |  |  |  |  |  |
| Recipient: No status info | -1.675 (-1.09) | -1.102 (-0.74) | -1.053 (-0.71) | -0.938 (-0.63) | -2.831 (-1.14) | -1.184 (-0.79) | -0.999 (-0.67) | -0.965 (-0.65) | -0.997 (-0.67) | -1.070 (-0.72) |
|  |  |  |  |  |  |  |  |  |  |  |
| Recipient: Middle status | -4.934** (-3.18) | -3.840* (-2.56) | -3.806* (-2.54) | -3.766* (-2.51) | -3.503 (-1.38) | -3.708* (-2.45) | -3.801* (-2.54) | -3.779* (-2.52) | -3.800* (-2.54) | -3.825* (-2.55) |
|  |  |  |  |  |  |  |  |  |  |  |
| Recipient: High status | -6.115*** (-3.99) | -5.729*** (-3.88) | -5.780*** (-3.91) | -5.681*** (-3.85) | -3.909 (-1.58) | -6.050*** (-4.06) | -5.732*** (-3.88) | -5.739*** (-3.89) | -5.689*** (-3.85) | -5.770*** (-3.91) |
|  |  |  |  |  |  |  |  |  |  |  |
| Age | -0.0494 (-1.50) | -0.0570+ (-1.76) | -0.0363 (-1.15) | -0.0459 (-1.45) | -0.00180 (-0.03) | -0.0314 (-0.98) | -0.0489 (-1.54) | -0.0405 (-1.28) | -0.0555+ (-1.73) | -0.0467 (-1.47) |
|  |  |  |  |  |  |  |  |  |  |  |
| Male | 0.213 (0.19) | -0.121 (-0.11) | 0.135 (0.13) | -0.0839 (-0.08) | 1.499 (0.83) | 0.213 (0.20) | -0.0247 (-0.02) | 0.0559 (0.05) | -0.152 (-0.14) | 0.0396 (0.04) |
|  |  |  |  |  |  |  |  |  |  |  |
| Education |  | -1.218* (-2.39) |  |  |  |  |  |  |  |  |
|  |  |  |  |  |  |  |  |  |  |  |
| Income deciles |  |  | -0.275 (-1.47) |  |  |  |  |  |  |  |
|  |  |  |  |  |  |  |  |  |  |  |
| Job prestige |  |  |  | -0.0871* (-2.27) |  |  |  |  |  |  |
|  |  |  |  |  |  |  |  |  |  |  |
| Job prestige (no imputations) |  |  |  |  | -0.193** (-3.01) |  |  |  |  |  |
|  |  |  |  |  |  |  |  |  |  |  |
| Income deciles (no imputations) |  |  |  |  |  | -0.321+ (-1.69) |  |  |  |  |
|  |  |  |  |  |  |  |  |  |  |  |
| Social status (composite: education, income and job prestige) |  |  |  |  |  |  | -1.444** (-2.69) |  |  |  |
|  |  |  |  |  |  |  |  |  |  |  |
| Social status (composite: income and job prestige) |  |  |  |  |  |  |  | -1.252* (-2.35) |  |  |
|  |  |  |  |  |  |  |  |  |  |  |
| Social status (composite: education and job prestige) |  |  |  |  |  |  |  |  | -1.476** (-2.71) |  |
|  |  |  |  |  |  |  |  |  |  |  |
| Social status (composite: education and income) |  |  |  |  |  |  |  |  |  | -1.256* (-2.34) |
|  |  |  |  |  |  |  |  |  |  |  |
| Constant | 56.76*** (28.34) | 61.46*** (21.11) | 57.55*** (27.10) | 60.26*** (23.06) | 61.66*** (13.79) | 57.58*** (26.96) | 56.73*** (29.35) | 56.26*** (29.22) | 57.10*** (29.28) | 56.62*** (29.31) |
| selection_ll |  |  |  |  |  |  |  |  |  |  |
| Objective social status (composite, cent.) | 0.0176 (0.27) |  |  |  |  |  |  |  |  |  |
|  |  |  |  |  |  |  |  |  |  |  |
| Recipient: No status info | -0.343* (-2.21) | -0.352* (-2.41) | -0.359* (-2.48) | -0.357* (-2.46) | -0.348 (-1.45) | -0.358* (-2.46) | -0.360* (-2.48) | -0.359* (-2.47) | -0.360* (-2.48) | -0.357* (-2.46) |
|  |  |  |  |  |  |  |  |  |  |  |
| Recipient: Middle status | -0.323* (-2.07) | -0.297* (-2.00) | -0.299* (-2.02) | -0.297* (-2.01) | -0.351 (-1.43) | -0.238 (-1.57) | -0.300* (-2.02) | -0.298* (-2.01) | -0.298* (-2.01) | -0.302* (-2.04) |
|  |  |  |  |  |  |  |  |  |  |  |
| Recipient: High status | -0.282+ (-1.81) | -0.210 (-1.40) | -0.209 (-1.40) | -0.208 (-1.40) | -0.181 (-0.73) | -0.208 (-1.39) | -0.211 (-1.41) | -0.209 (-1.40) | -0.212 (-1.42) | -0.208 (-1.39) |
|  |  |  |  |  |  |  |  |  |  |  |
| Age | -0.00116 (-0.37) | 0.000590 (0.20) | -0.000728 (-0.25) | -0.000743 (-0.25) | -0.00146 (-0.29) | -0.00153 (-0.51) | -0.000422 (-0.14) | -0.000693 (-0.24) | -0.000176 (-0.06) | -0.000290 (-0.10) |
|  |  |  |  |  |  |  |  |  |  |  |
| Male | -0.322** (-3.04) | -0.319** (-3.17) | -0.327** (-3.26) | -0.326** (-3.26) | -0.433* (-2.52) | -0.325** (-3.20) | -0.325** (-3.24) | -0.325** (-3.25) | -0.322** (-3.21) | -0.328** (-3.26) |
|  |  |  |  |  |  |  |  |  |  |  |
| Education |  | 0.108* (2.28) |  |  |  |  |  |  |  |  |
|  |  |  |  |  |  |  |  |  |  |  |
| Income deciles |  |  | 0.00431 (0.25) |  |  |  |  |  |  |  |
|  |  |  |  |  |  |  |  |  |  |  |
| Job prestige |  |  |  | -0.000920 (-0.26) |  |  |  |  |  |  |
|  |  |  |  |  |  |  |  |  |  |  |
| Job prestige (no imputations) |  |  |  |  | -0.00568 (-0.98) |  |  |  |  |  |
|  |  |  |  |  |  |  |  |  |  |  |
| Income deciles (no imputations) |  |  |  |  |  | 0.00430 (0.24) |  |  |  |  |
|  |  |  |  |  |  |  |  |  |  |  |
| Social status (composite: education, income and job prestige) |  |  |  |  |  |  | 0.0490 (0.98) |  |  |  |
|  |  |  |  |  |  |  |  |  |  |  |
| Social status (composite: income and job prestige) |  |  |  |  |  |  |  | -0.000597 (-0.01) |  |  |
|  |  |  |  |  |  |  |  |  |  |  |
| Social status (composite: education and job prestige) |  |  |  |  |  |  |  |  | 0.0581 (1.15) |  |
|  |  |  |  |  |  |  |  |  |  |  |
| Social status (composite: education and income) |  |  |  |  |  |  |  |  |  | 0.0775 (1.54) |
|  |  |  |  |  |  |  |  |  |  |  |
| Constant | 2.148*** (10.40) | 1.669*** (6.25) | 2.083*** (9.95) | 2.146*** (8.64) | 2.408*** (5.48) | 2.113*** (9.96) | 2.094*** (10.87) | 2.104*** (10.95) | 2.081*** (10.75) | 2.091*** (10.85) |
| lnsigma |  |  |  |  |  |  |  |  |  |  |
| Constant | 3.085*** (158.42) | 3.101*** (167.27) | 3.102*** (167.27) | 3.101*** (167.27) | 3.078*** (97.26) | 3.102*** (165.94) | 3.100*** (167.28) | 3.101*** (167.27) | 3.100*** (167.28) | 3.101*** (167.27) |
| Observations | 1766 | 1962 | 1962 | 1962 | 666 | 1930 | 1962 | 1962 | 1962 | 1962 |
| Log likelihood | -7833.5 | -8727.5 | -8731.9 | -8730.4 | -2943.8 | -8591.7 | -8728.9 | -8730.2 | -8728.6 | -8729.0 |

Note: z statistics in parentheses; + p<0.10, * p<0.05, ** p<0.01, *** p<0.001.

**Table S11.** Additional robustness analyses for Poland; Cragg Hurdle Models on the percentage of time kept in the TDG

|  | M1 | M2 | M3 | M4 | M5 | M6 | M7 | M8 | M9 | M10 |
| --- | --- | --- | --- | --- | --- | --- | --- | --- | --- | --- |
| Percentage kept TDG |  |  |  |  |  |  |  |  |  |  |
| Objective social status (composite, cent.) | -0.688 (-0.72) |  |  |  |  |  |  |  |  |  |
|  |  |  |  |  |  |  |  |  |  |  |
| Recipient: No status info | 1.536 (0.72) | 1.668 (0.82) | 1.751 (0.86) | 1.695 (0.84) | 5.179+ (1.72) | 1.791 (0.88) | 1.646 (0.81) | 1.663 (0.82) | 1.689 (0.83) | 1.652 (0.82) |
|  |  |  |  |  |  |  |  |  |  |  |
| Recipient: Middle status | -1.531 (-0.72) | -1.537 (-0.76) | -1.387 (-0.68) | -1.474 (-0.73) | -2.124 (-0.69) | -1.521 (-0.75) | -1.510 (-0.74) | -1.467 (-0.72) | -1.523 (-0.75) | -1.485 (-0.73) |
|  |  |  |  |  |  |  |  |  |  |  |
| Recipient: High status | -3.700+ (-1.72) | -2.263 (-1.11) | -2.080 (-1.02) | -2.154 (-1.06) | 1.406 (0.45) | -2.202 (-1.08) | -2.203 (-1.08) | -2.153 (-1.06) | -2.230 (-1.10) | -2.176 (-1.07) |
|  |  |  |  |  |  |  |  |  |  |  |
| Age | -0.0420 (-0.79) | -0.0355 (-0.72) | -0.0694 (-1.42) | -0.0357 (-0.72) | -0.0111 (-0.15) | -0.0680 (-1.38) | -0.0399 (-0.79) | -0.0533 (-1.06) | -0.0260 (-0.52) | -0.0508 (-1.02) |
|  |  |  |  |  |  |  |  |  |  |  |
| Male | -3.138* (-2.05) | -2.897* (-2.00) | -3.303* (-2.26) | -2.880* (-1.99) | -0.00439 (-0.00) | -3.225* (-2.20) | -2.848+ (-1.95) | -2.990* (-2.05) | -2.820+ (-1.95) | -2.955* (-2.03) |
|  |  |  |  |  |  |  |  |  |  |  |
| Education |  | -1.434* (-1.98) |  |  |  |  |  |  |  |  |
|  |  |  |  |  |  |  |  |  |  |  |
| Income deciles |  |  | 0.330 (1.26) |  |  |  |  |  |  |  |
|  |  |  |  |  |  |  |  |  |  |  |
| Job prestige |  |  |  | -0.0861 (-1.61) |  |  |  |  |  |  |
|  |  |  |  |  |  |  |  |  |  |  |
| Job prestige (no imputations) |  |  |  |  | -0.169* (-2.15) |  |  |  |  |  |
|  |  |  |  |  |  |  |  |  |  |  |
| Income deciles (no imputations) |  |  |  |  |  | 0.327 (1.24) |  |  |  |  |
|  |  |  |  |  |  |  |  |  |  |  |
| Social status (composite: education, income and job prestige) |  |  |  |  |  |  | -0.825 (-1.07) |  |  |  |
|  |  |  |  |  |  |  |  |  |  |  |
| Social status (composite: income and job prestige) |  |  |  |  |  |  |  | -0.170 (-0.22) |  |  |
|  |  |  |  |  |  |  |  |  |  |  |
| Social status (composite: education and job prestige) |  |  |  |  |  |  |  |  | -1.629* (-2.16) |  |
|  |  |  |  |  |  |  |  |  |  |  |
| Social status (composite: education and income) |  |  |  |  |  |  |  |  |  | -0.342 (-0.45) |
|  |  |  |  |  |  |  |  |  |  |  |
| Constant | 65.54*** (24.04) | 69.59*** (20.93) | 64.08*** (23.89) | 68.02*** (22.41) | 67.80*** (14.61) | 64.15*** (23.84) | 64.51*** (24.74) | 65.11*** (24.99) | 63.91*** (24.72) | 65.00*** (25.20) |
| selection_ll |  |  |  |  |  |  |  |  |  |  |
| Objective social status (composite, cent.) | 0.0444 (0.78) |  |  |  |  |  |  |  |  |  |
|  |  |  |  |  |  |  |  |  |  |  |
| Recipient: No status info | -0.0559 (-0.45) | -0.143 (-1.23) | -0.141 (-1.21) | -0.143 (-1.23) | -0.275 (-1.50) | -0.159 (-1.35) | -0.143 (-1.23) | -0.142 (-1.22) | -0.143 (-1.23) | -0.143 (-1.23) |
|  |  |  |  |  |  |  |  |  |  |  |
| Recipient: Middle status | 0.0751 (0.58) | 0.0988 (0.79) | 0.0988 (0.79) | 0.100 (0.80) | 0.0869 (0.42) | 0.0811 (0.64) | 0.0994 (0.79) | 0.100 (0.80) | 0.0990 (0.79) | 0.0990 (0.79) |
|  |  |  |  |  |  |  |  |  |  |  |
| Recipient: High status | -0.0105 (-0.08) | 0.0220 (0.18) | 0.0266 (0.22) | 0.0253 (0.21) | -0.0499 (-0.25) | 0.0397 (0.32) | 0.0256 (0.21) | 0.0275 (0.22) | 0.0239 (0.20) | 0.0247 (0.20) |
|  |  |  |  |  |  |  |  |  |  |  |
| Age | 0.00278 (0.85) | 0.00347 (1.14) | 0.00273 (0.90) | 0.00285 (0.93) | 0.00189 (0.39) | 0.00301 (0.98) | 0.00286 (0.92) | 0.00249 (0.80) | 0.00317 (1.03) | 0.00305 (0.99) |
|  |  |  |  |  |  |  |  |  |  |  |
| Male | -0.110 (-1.21) | -0.120 (-1.40) | -0.132 (-1.52) | -0.126 (-1.47) | -0.215 (-1.61) | -0.125 (-1.43) | -0.127 (-1.47) | -0.133 (-1.54) | -0.123 (-1.43) | -0.125 (-1.45) |
|  |  |  |  |  |  |  |  |  |  |  |
| Education |  | -0.0179 (-0.41) |  |  |  |  |  |  |  |  |
|  |  |  |  |  |  |  |  |  |  |  |
| Income deciles |  |  | 0.0115 (0.74) |  |  |  |  |  |  |  |
|  |  |  |  |  |  |  |  |  |  |  |
| Job prestige |  |  |  | 0.00157 (0.49) |  |  |  |  |  |  |
|  |  |  |  |  |  |  |  |  |  |  |
| Job prestige (no imputations) |  |  |  |  | 0.00491 (1.00) |  |  |  |  |  |
|  |  |  |  |  |  |  |  |  |  |  |
| Income deciles (no imputations) |  |  |  |  |  | 0.00513 (0.33) |  |  |  |  |
|  |  |  |  |  |  |  |  |  |  |  |
| Social status (composite: education, income and job prestige) |  |  |  |  |  |  | 0.0169 (0.37) |  |  |  |
|  |  |  |  |  |  |  |  |  |  |  |
| Social status (composite: income and job prestige) |  |  |  |  |  |  |  | 0.0368 (0.80) |  |  |
|  |  |  |  |  |  |  |  |  |  |  |
| Social status (composite: education and job prestige) |  |  |  |  |  |  |  |  | 0.00186 (0.04) |  |
|  |  |  |  |  |  |  |  |  |  |  |
| Social status (composite: education and income) |  |  |  |  |  |  |  |  |  | 0.00894 (0.20) |
|  |  |  |  |  |  |  |  |  |  |  |
| Constant | 1.382*** (8.32) | 1.429*** (7.14) | 1.337*** (8.31) | 1.324*** (7.17) | 1.361*** (4.54) | 1.366*** (8.39) | 1.391*** (8.79) | 1.410*** (8.90) | 1.377*** (8.78) | 1.383*** (8.81) |
| lnsigma |  |  |  |  |  |  |  |  |  |  |
| Constant | 3.360*** (161.21) | 3.359*** (169.25) | 3.359*** (169.23) | 3.359*** (169.24) | 3.338*** (111.70) | 3.358*** (168.37) | 3.360*** (169.23) | 3.360*** (169.22) | 3.358*** (169.25) | 3.360*** (169.22) |
| Observations | 1733 | 1924 | 1924 | 1924 | 826 | 1899 | 1924 | 1924 | 1924 | 1924 |
| Log likelihood | -8023.6 | -8897.0 | -8898.0 | -8897.6 | -3808.5 | -8783.4 | -8898.4 | -8898.7 | -8896.7 | -8898.9 |

Note: z statistics in parentheses; + p<0.10, * p<0.05, ** p<0.01, *** p<0.001.

**Table S12.** Additional robustness analyses for Sweden; Cragg Hurdle Models on the percentage of time kept in the TDG

|  | M1 | M2 | M3 | M4 | M10 |
| --- | --- | --- | --- | --- | --- |
| Percentage kept TDG |  |  |  |  |  |
| Objective social status (composite, cent.) | -2.333*** (-3.79) |  |  |  |  |
|  |  |  |  |  |  |
| Recipient: No status info | 1.977 (1.36) | 1.271 (0.90) | 1.076 (0.76) | 1.027 (0.72) | 1.189 (0.85) |
|  |  |  |  |  |  |
| Recipient: Middle status | 2.251 (1.59) | 1.568 (1.14) | 1.519 (1.10) | 1.161 (0.83) | 1.628 (1.18) |
|  |  |  |  |  |  |
| Recipient: High status | 1.181 (0.83) | 0.865 (0.62) | 0.827 (0.60) | 0.511 (0.36) | 0.945 (0.68) |
|  |  |  |  |  |  |
| Age | -0.136*** (-4.29) | -0.122*** (-4.08) | -0.108*** (-3.62) | -0.112*** (-3.67) | -0.116*** (-3.91) |
|  |  |  |  |  |  |
| Male | 1.873+ (1.65) | 0.901 (0.84) | 1.087 (1.00) | 1.089 (0.99) | 1.216 (1.12) |
|  |  |  |  |  |  |
| Education |  | -1.313** (-3.21) |  |  |  |
|  |  |  |  |  |  |
| Income deciles |  |  | -0.502** (-2.82) |  |  |
|  |  |  |  |  |  |
| Income deciles (no imputations) |  |  |  | -0.486** (-2.69) |  |
|  |  |  |  |  |  |
| Social status (composite: education and income) |  |  |  |  | -1.954*** (-3.85) |
|  |  |  |  |  |  |
| Constant | 54.46*** (31.46) | 59.44*** (25.96) | 56.68*** (30.62) | 57.08*** (30.41) | 54.21*** (32.67) |
| selection_ll |  |  |  |  |  |
| Objective social status (composite, cent.) | 0.0668 (1.10) |  |  |  |  |
|  |  |  |  |  |  |
| Recipient: No status info | -0.569*** (-3.61) | -0.531*** (-3.52) | -0.530*** (-3.51) | -0.504*** (-3.30) | -0.532*** (-3.52) |
|  |  |  |  |  |  |
| Recipient: Middle status | -0.486** (-3.06) | -0.445** (-2.93) | -0.449** (-2.96) | -0.434** (-2.84) | -0.448** (-2.95) |
|  |  |  |  |  |  |
| Recipient: High status | -0.288+ (-1.72) | -0.252 (-1.57) | -0.259 (-1.61) | -0.245 (-1.51) | -0.256 (-1.60) |
|  |  |  |  |  |  |
| Age | 0.00385 (1.25) | 0.00440 (1.51) | 0.00415 (1.42) | 0.00319 (1.07) | 0.00433 (1.49) |
|  |  |  |  |  |  |
| Male | -0.304** (-2.72) | -0.256* (-2.40) | -0.272* (-2.52) | -0.250* (-2.27) | -0.264* (-2.46) |
|  |  |  |  |  |  |
| Education |  | -0.00331 (-0.08) |  |  |  |
|  |  |  |  |  |  |
| Income deciles |  |  | 0.0169 (0.97) |  |  |
|  |  |  |  |  |  |
| Income deciles (no imputations) |  |  |  | 0.0208 (1.17) |  |
|  |  |  |  |  |  |
| Social status (composite: education and income) |  |  |  |  | 0.0292 (0.58) |
|  |  |  |  |  |  |
| Constant | 1.952*** (10.19) | 1.907*** (8.18) | 1.825*** (9.43) | 1.829*** (9.34) | 1.904*** (10.50) |
| lnsigma |  |  |  |  |  |
| Constant | 3.007*** (156.37) | 3.021*** (163.91) | 3.022*** (163.91) | 3.020*** (161.64) | 3.020*** (163.90) |
| Observations | 1743 | 1911 | 1911 | 1853 | 1911 |
| Log likelihood | -7600.9 | -8365.7 | -8366.4 | -8114.0 | -8363.3 |

Note: z statistics in parentheses; + p<0.10, * p<0.05, ** p<0.01, *** p<0.001.

**Table S13.** Additional robustness analyses for USA; Cragg Hurdle Models on the percentage of time kept in the TDG

|  | M1 | M2 | M3 | M4 | M5 | M6 | M7 | M8 | M9 | M10 |
| --- | --- | --- | --- | --- | --- | --- | --- | --- | --- | --- |
| Percentage kept TDG |  |  |  |  |  |  |  |  |  |  |
| Objective social status (composite, cent.) | -3.268*** (-3.79) |  |  |  |  |  |  |  |  |  |
|  |  |  |  |  |  |  |  |  |  |  |
| Recipient: No status info | 1.522 (0.77) | 0.927 (0.50) | 0.753 (0.40) | 0.653 (0.35) | -3.652 (-1.11) | 0.161 (0.09) | 0.791 (0.42) | 0.675 (0.36) | 0.773 (0.41) | 0.906 (0.49) |
|  |  |  |  |  |  |  |  |  |  |  |
| Recipient: Middle status | -0.955 (-0.48) | -1.728 (-0.92) | -1.651 (-0.88) | -1.916 (-1.02) | -3.567 (-1.05) | -1.870 (-1.00) | -1.808 (-0.97) | -1.845 (-0.99) | -1.879 (-1.00) | -1.643 (-0.88) |
|  |  |  |  |  |  |  |  |  |  |  |
| Recipient: High status | -2.503 (-1.26) | -2.346 (-1.24) | -2.508 (-1.33) | -2.440 (-1.29) | -1.988 (-0.57) | -2.531 (-1.34) | -2.356 (-1.25) | -2.427 (-1.28) | -2.351 (-1.24) | -2.382 (-1.26) |
|  |  |  |  |  |  |  |  |  |  |  |
| Age | -0.0647 (-1.64) | -0.0679+ (-1.81) | -0.0734* (-1.97) | -0.0752* (-2.00) | 0.0124 (0.18) | -0.0627+ (-1.68) | -0.0615 (-1.64) | -0.0669+ (-1.78) | -0.0667+ (-1.78) | -0.0629+ (-1.68) |
|  |  |  |  |  |  |  |  |  |  |  |
| Male | -1.691 (-1.19) | -1.870 (-1.39) | -1.287 (-0.96) | -1.306 (-0.97) | 0.167 (0.07) | -1.040 (-0.78) | -1.737 (-1.30) | -1.418 (-1.06) | -1.688 (-1.26) | -1.806 (-1.35) |
|  |  |  |  |  |  |  |  |  |  |  |
| Education |  | -2.260*** (-4.06) |  |  |  |  |  |  |  |  |
|  |  |  |  |  |  |  |  |  |  |  |
| Income deciles |  |  | -0.889*** (-3.81) |  |  |  |  |  |  |  |
|  |  |  |  |  |  |  |  |  |  |  |
| Job prestige |  |  |  | -0.113* (-2.32) |  |  |  |  |  |  |
|  |  |  |  |  |  |  |  |  |  |  |
| Job prestige (no imputations) |  |  |  |  | -0.116 (-1.29) |  |  |  |  |  |
|  |  |  |  |  |  |  |  |  |  |  |
| Income deciles (no imputations) |  |  |  |  |  | -1.001*** (-4.29) |  |  |  |  |
|  |  |  |  |  |  |  |  |  |  |  |
| Social status (composite: education, income and job prestige) |  |  |  |  |  |  | -3.084*** (-4.58) |  |  |  |
|  |  |  |  |  |  |  |  |  |  |  |
| Social status (composite: income and job prestige) |  |  |  |  |  |  |  | -2.633*** (-3.92) |  |  |
|  |  |  |  |  |  |  |  |  |  |  |
| Social status (composite: education and job prestige) |  |  |  |  |  |  |  |  | -2.514*** (-3.72) |  |
|  |  |  |  |  |  |  |  |  |  |  |
| Social status (composite: education and income) |  |  |  |  |  |  |  |  |  | -3.310*** (-4.90) |
|  |  |  |  |  |  |  |  |  |  |  |
| Constant | 54.59*** (23.44) | 63.26*** (21.83) | 59.89*** (24.23) | 59.96*** (20.59) | 57.23*** (10.20) | 60.00*** (24.25) | 54.66*** (24.77) | 54.80*** (24.79) | 54.89*** (24.84) | 54.73*** (24.85) |
| selection_ll |  |  |  |  |  |  |  |  |  |  |
| Objective social status (composite, cent.) | -0.0932+ (-1.79) |  |  |  |  |  |  |  |  |  |
|  |  |  |  |  |  |  |  |  |  |  |
| Recipient: No status info | -0.234* (-2.00) | -0.151 (-1.37) | -0.152 (-1.39) | -0.150 (-1.36) | -0.0493 (-0.24) | -0.158 (-1.43) | -0.151 (-1.37) | -0.151 (-1.38) | -0.150 (-1.36) | -0.152 (-1.38) |
|  |  |  |  |  |  |  |  |  |  |  |
| Recipient: Middle status | -0.0597 (-0.49) | 0.0204 (0.18) | 0.0230 (0.20) | 0.0187 (0.16) | 0.133 (0.59) | 0.0505 (0.43) | 0.0207 (0.18) | 0.0221 (0.19) | 0.0183 (0.16) | 0.0219 (0.19) |
|  |  |  |  |  |  |  |  |  |  |  |
| Recipient: High status | 0.0250 (0.20) | 0.0407 (0.35) | 0.0426 (0.37) | 0.0472 (0.40) | 0.271 (1.14) | 0.0310 (0.27) | 0.0444 (0.38) | 0.0467 (0.40) | 0.0432 (0.37) | 0.0422 (0.36) |
|  |  |  |  |  |  |  |  |  |  |  |
| Age | 0.00346 (1.40) | 0.00234 (1.00) | 0.00214 (0.92) | 0.00248 (1.06) | 0.00639 (1.34) | 0.00180 (0.76) | 0.00269 (1.15) | 0.00259 (1.11) | 0.00266 (1.14) | 0.00240 (1.03) |
|  |  |  |  |  |  |  |  |  |  |  |
| Male | -0.242** (-2.81) | -0.205* (-2.54) | -0.193* (-2.41) | -0.203* (-2.52) | -0.186 (-1.17) | -0.173* (-2.13) | -0.206* (-2.55) | -0.198* (-2.46) | -0.211** (-2.61) | -0.200* (-2.49) |
|  |  |  |  |  |  |  |  |  |  |  |
| Education |  | -0.0450 (-1.35) |  |  |  |  |  |  |  |  |
|  |  |  |  |  |  |  |  |  |  |  |
| Income deciles |  |  | -0.0103 (-0.75) |  |  |  |  |  |  |  |
|  |  |  |  |  |  |  |  |  |  |  |
| Job prestige |  |  |  | -0.00861** (-2.95) |  |  |  |  |  |  |
|  |  |  |  |  |  |  |  |  |  |  |
| Job prestige (no imputations) |  |  |  |  | -0.0117* (-2.06) |  |  |  |  |  |
|  |  |  |  |  |  |  |  |  |  |  |
| Income deciles (no imputations) |  |  |  |  |  | -0.00965 (-0.69) |  |  |  |  |
|  |  |  |  |  |  |  |  |  |  |  |
| Social status (composite: education, income and job prestige) |  |  |  |  |  |  | -0.0928* (-2.28) |  |  |  |
|  |  |  |  |  |  |  |  |  |  |  |
| Social status (composite: income and job prestige) |  |  |  |  |  |  |  | -0.0948* (-2.36) |  |  |
|  |  |  |  |  |  |  |  |  |  |  |
| Social status (composite: education and job prestige) |  |  |  |  |  |  |  |  | -0.103* (-2.52) |  |
|  |  |  |  |  |  |  |  |  |  |  |
| Social status (composite: education and income) |  |  |  |  |  |  |  |  |  | -0.0534 (-1.32) |
|  |  |  |  |  |  |  |  |  |  |  |
| Constant | 1.380*** (9.41) | 1.515*** (8.46) | 1.408*** (9.32) | 1.716*** (9.31) | 1.674*** (4.40) | 1.412*** (9.24) | 1.337*** (9.75) | 1.337*** (9.75) | 1.343*** (9.80) | 1.345*** (9.81) |
| lnsigma |  |  |  |  |  |  |  |  |  |  |
| Constant | 3.259*** (147.23) | 3.255*** (154.99) | 3.256*** (154.97) | 3.259*** (154.88) | 3.237*** (84.34) | 3.248*** (153.84) | 3.254*** (155.04) | 3.256*** (154.98) | 3.256*** (154.97) | 3.253*** (155.07) |
| Observations | 1734 | 1925 | 1925 | 1925 | 554 | 1893 | 1925 | 1925 | 1925 | 1925 |
| Log likelihood | -7746.2 | -8593.0 | -8594.6 | -8595.1 | -2480.2 | -8445.7 | -8589.1 | -8591.7 | -8592.0 | -8589.3 |

Note: z statistics in parentheses; + p<0.10, * p<0.05, ** p<0.01, *** p<0.001.

**Table S14.** Cragg Hurdle Models on the percentage of money shared in the MDG, seemingly unrelated regression models

|  | (1)  Pooled model | (2)  Pooled model | (3)  Germany | (4)  Germany | (5)  Poland | (6)  Poland | (7)  Sweden | (8)  Sweden | (9)  USA | (10)  USA |
| --- | --- | --- | --- | --- | --- | --- | --- | --- | --- | --- |
|  | Objective  SES | Subjective  SES | Objective  SES | Subjective SES | Objective SES | Subjective SES | Objective  SES | Subjective  SES | Objective  SES | Subjective SES |
| Percentage given MDG |  |  |  |  |  |  |  |  |  |  |
| Social status (composite, cent.) | 2.473*** (6.90) |  | 1.496* (2.53) |  | 1.906 (1.64) |  | 2.878*** (4.76) |  | 3.740*** (4.46) |  |
|  |  |  |  |  |  |  |  |  |  |  |
| Subjective social status |  | 2.290*** (20.42) |  | 1.785*** (5.45) |  | 2.637*** (4.60) |  | 2.232^***^ (7.33) |  | 2.871*** (7.72) |
|  |  |  |  |  |  |  |  |  |  |  |
| Recipient: No status info | -6.736*** (-5.38) | -6.609*** (-4.52) | -5.180** (-3.21) | -4.689** (-2.93) | -5.271+ (-1.77) | -5.268+ (-1.81) | -5.898*** (-3.52) | -5.704*** (-3.44) | -11.15*** (-4.77) | -11.84*** (-5.18) |
|  |  |  |  |  |  |  |  |  |  |  |
| Recipient: Middle status | -5.167*** (-8.03) | -5.303*** (-7.83) | -4.036** (-2.76) | -4.077** (-2.82) | -4.248 (-1.49) | -4.329 (-1.55) | -6.412*** (-4.13) | -6.355*** (-4.19) | -6.323** (-3.13) | -6.974*** (-3.54) |
|  |  |  |  |  |  |  |  |  |  |  |
| Recipient: High status | -9.151*** (-5.43) | -9.187*** (-5.55) | -7.020*** (-4.37) | -6.748*** (-4.26) | -5.994* (-2.01) | -6.444* (-2.21) | -13.07*** (-7.79) | -13.01*** (-7.86) | -9.984*** (-4.48) | -10.28*** (-4.76) |
|  |  |  |  |  |  |  |  |  |  |  |
| Age | 0.00777 (0.16) | 0.00795 (0.15) | 0.00382 (0.12) | -0.00354 (-0.11) | 0.0683 (0.98) | 0.100 (1.55) | 0.0273 (0.83) | 0.0161 (0.50) | -0.110* (-2.53) | -0.100* (-2.39) |
|  |  |  |  |  |  |  |  |  |  |  |
| Male | 2.909* (2.54) | 2.533+ (1.95) | 0.902 (0.81) | 0.778 (0.70) | 6.744** (3.12) | 7.078*** (3.35) | 0.763 (0.61) | -0.0501 (-0.04) | 4.928** (3.08) | 3.901* (2.55) |
|  |  |  |  |  |  |  |  |  |  |  |
| Constant | 46.89*** (9.22) | 35.78*** (6.72) | 46.96*** (23.65) | 38.26*** (14.98) | 29.27*** (7.11) | 14.92** (2.90) | 53.99*** (27.15) | 43.62*** (18.85) | 53.88*** (20.84) | 40.81*** (13.25) |
| Given MDG > 0 |  |  |  |  |  |  |  |  |  |  |
| Social status (composite, cent.) | 0.0153 (1.07) |  | 0.0273 (0.63) |  | 0.0196 (0.56) |  | 0.0522 (1.24) |  | -0.0259 (-0.67) |  |
|  |  |  |  |  |  |  |  |  |  |  |
| Subjective social status |  | 0.0371* (2.40) |  | 0.0260 (1.19) |  | 0.0161 (1.02) |  | 0.0871*** (4.27) |  | 0.0422* (2.41) |
|  |  |  |  |  |  |  |  |  |  |  |
| Recipient: No status info | -0.303*** (-9.38) | -0.300*** (-8.70) | -0.349** (-3.01) | -0.341** (-2.93) | -0.268** (-2.93) | -0.268** (-2.93) | -0.236+ (-1.88) | -0.222+ (-1.75) | -0.384*** (-3.49) | -0.400*** (-3.62) |
|  |  |  |  |  |  |  |  |  |  |  |
| Recipient: Middle status | -0.112* (-1.96) | -0.113* (-1.98) | -0.0681 (-0.56) | -0.0681 (-0.56) | -0.0393 (-0.41) | -0.0392 (-0.41) | -0.305* (-2.51) | -0.296* (-2.42) | -0.127 (-1.09) | -0.142 (-1.22) |
|  |  |  |  |  |  |  |  |  |  |  |
| Recipient: High status | -0.242*** (-4.41) | -0.240*** (-4.47) | -0.252* (-2.13) | -0.237* (-2.00) | -0.144 (-1.54) | -0.147 (-1.57) | -0.370** (-3.10) | -0.360** (-2.98) | -0.298** (-2.64) | -0.314** (-2.79) |
|  |  |  |  |  |  |  |  |  |  |  |
| Age | 0.00891*** (9.04) | 0.00892*** (8.36) | 0.00689** (2.99) | 0.00700** (3.03) | 0.00596* (2.56) | 0.00637** (2.89) | 0.0101*** (4.27) | 0.00953*** (3.92) | 0.00653** (2.99) | 0.00613** (2.79) |
|  |  |  |  |  |  |  |  |  |  |  |
| Male | -0.0862* (-2.11) | -0.0947* (-2.04) | -0.142+ (-1.73) | -0.149+ (-1.81) | 0.00641 (0.10) | 0.00912 (0.14) | -0.140 (-1.59) | -0.192* (-2.19) | -0.153* (-1.98) | -0.151* (-1.96) |
|  |  |  |  |  |  |  |  |  |  |  |
| Constant | 0.980*** (5.17) | 0.805*** (5.26) | 1.284*** (8.71) | 1.152*** (6.38) | 0.702*** (5.81) | 0.603*** (4.43) | 1.198*** (8.63) | 0.826*** (4.93) | 1.222*** (9.18) | 1.058*** (7.10) |
| lnsigma |  |  |  |  |  |  |  |  |  |  |
| Constant | 3.265*** (954.55) | 3.251*** (947.05) | 3.092*** (2695.18) | 3.082*** (2677.21) | 3.479*** (3650.09) | 3.461*** (3599.13) | 3.113*** (2985.61) | 3.100*** (2894.62) | 3.352*** (3991.99) | 3.333*** (3869.25) |
| Observations | 7722 | 7718 | 1962 | 1961 | 1924 | 1922 | 1911 | 1910 | 1925 | 1925 |

Note: z statistics in parentheses; + p<0.10, * p<0.05, ** p<0.01, *** p<0.001.

**Table S15.** Cragg Hurdle Models on the percentage of time kept in the TDG, seemingly unrelated regression models

|  | (11)  Pooled model | (12)  Pooled model | (13)  Germany | (14) Germany | (15)  Poland | (16)  Poland | (17)  Sweden | (18)  Sweden | (19)  USA | (20)  USA |
| --- | --- | --- | --- | --- | --- | --- | --- | --- | --- | --- |
|  | Objective  SES | Subjective  SES | Objective  SES | Subjective SES | Objective  SES | Subjective SES | Objective  SES | Subjective  SES | Objective  SES | Subjective SES |
| Percentage kept TDG |  |  |  |  |  |  |  |  |  |  |
| Social status (composite, cent.) | -1.713*** (-3.36) |  | -1.444* (-2.52) |  | -0.825 (-1.08) |  | -1.954*** (-3.74) |  | -3.084*** (-4.55) |  |
|  |  |  |  |  |  |  |  |  |  |  |
| Subjective social status |  | -0.0307** (-2.59) |  | -1.442*** (-4.88) |  | -1.483*** (-4.02) |  | -1.857*** (-6.90) |  | -2.108*** (-6.60) |
|  |  |  |  |  |  |  |  |  |  |  |
| Recipient: No status info | 0.813 (1.16) | -0.256** (-3.18) | -0.999 (-0.66) | -1.297 (-0.86) | 1.646 (0.81) | 1.505 (0.75) | 1.189 (0.82) | 1.120 (0.78) | 0.791 (0.42) | 0.675 (0.36) |
|  |  |  |  |  |  |  |  |  |  |  |
| Recipient: Middle status | -1.271 (-1.07) | -0.111 (-0.97) | -3.801** (-2.69) | -3.838** (-2.74) | -1.510 (-0.77) | -1.395 (-0.72) | 1.628 (1.26) | 1.650 (1.30) | -1.808 (-1.00) | -1.889 (-1.06) |
|  |  |  |  |  |  |  |  |  |  |  |
| Recipient: High status | -2.389 (-1.61) | -0.0592 (-0.89) | -5.732*** (-4.04) | -5.534*** (-3.93) | -2.203 (-1.10) | -2.260 (-1.13) | 0.945 (0.70) | 1.297 (0.97) | -2.356 (-1.26) | -2.578 (-1.39) |
|  |  |  |  |  |  |  |  |  |  |  |
| Age | -0.106*** (-4.15) | 0.00335*** (3.76) | -0.0489 (-1.60) | -0.0408 (-1.36) | -0.0399 (-0.80) | -0.0530 (-1.12) | -0.116*** (-3.98) | -0.104*** (-3.59) | -0.0615+ (-1.65) | -0.0700+ (-1.90) |
|  |  |  |  |  |  |  |  |  |  |  |
| Male | -0.717 (-0.93) | -0.206*** (-5.41) | -0.0247 (-0.02) | 0.0869 (0.08) | -2.848+ (-1.95) | -2.884* (-1.99) | 1.216 (1.11) | 2.048+ (1.89) | -1.737 (-1.29) | -1.043 (-0.79) |
|  |  |  |  |  |  |  |  |  |  |  |
| Constant | 59.08*** (15.37) | 1.722*** (17.11) | 56.73*** (30.81) | 63.53*** (27.48) | 64.51*** (25.47) | 72.47*** (24.56) | 54.21*** (33.31) | 62.36*** (30.89) | 54.66*** (25.20) | 64.71*** (26.78) |
| Kept TDG > 0 |  |  |  |  |  |  |  |  |  |  |
| Social status (composite, cent.) | -0.00682 (-0.19) |  | 0.0490 (0.95) |  | 0.0169 (0.37) |  | 0.0292 (0.60) |  | -0.0928* (-2.24) |  |
|  |  |  |  |  |  |  |  |  |  |  |
| Subjective social status |  | -0.0307** (-2.59) |  | 0.00996 (0.37) |  | -0.0481* (-2.11) |  | -0.0238 (-0.88) |  | -0.0495** (-2.61) |
|  |  |  |  |  |  |  |  |  |  |  |
| Recipient: No status info | -0.253** (-3.13) | -0.256** (-3.18) | -0.360* (-2.49) | -0.359* (-2.48) | -0.143 (-1.23) | -0.154 (-1.30) | -0.532*** (-3.55) | -0.534*** (-3.58) | -0.151 (-1.37) | -0.147 (-1.34) |
|  |  |  |  |  |  |  |  |  |  |  |
| Recipient: Middle status | -0.108 (-0.92) | -0.111 (-0.97) | -0.300* (-2.04) | -0.301* (-2.06) | 0.0994 (0.79) | 0.0966 (0.77) | -0.448** (-2.99) | -0.448** (-3.00) | 0.0207 (0.18) | 0.0143 (0.12) |
|  |  |  |  |  |  |  |  |  |  |  |
| Recipient: High status | -0.0576 (-0.84) | -0.0592 (-0.89) | -0.211 (-1.42) | -0.214 (-1.46) | 0.0256 (0.21) | 0.00829 (0.07) | -0.256 (-1.61) | -0.250 (-1.57) | 0.0444 (0.38) | 0.0395 (0.34) |
|  |  |  |  |  |  |  |  |  |  |  |
| Age | 0.00331*** (4.05) | 0.00335*** (3.76) | -0.000422 (-0.15) | -0.000678 (-0.24) | 0.00286 (1.03) | 0.00307 (1.17) | 0.00433 (1.43) | 0.00457 (1.50) | 0.00269 (1.27) | 0.00236 (1.11) |
|  |  |  |  |  |  |  |  |  |  |  |
| Male | -0.209*** (-5.35) | -0.206*** (-5.41) | -0.325** (-3.28) | -0.324** (-3.27) | -0.127 (-1.48) | -0.123 (-1.43) | -0.264* (-2.43) | -0.239* (-2.18) | -0.206* (-2.52) | -0.192* (-2.38) |
|  |  |  |  |  |  |  |  |  |  |  |
| Constant | 1.566*** (9.47) | 1.722*** (17.11) | 2.094*** (10.71) | 2.055*** (8.66) | 1.391*** (9.50) | 1.643*** (8.59) | 1.904*** (11.01) | 2.001*** (9.43) | 1.337*** (10.38) | 1.583*** (9.88) |
| lnsigma |  |  |  |  |  |  |  |  |  |  |
| Constant | 3.221*** (932.69) | 3.213*** (908.75) | 3.100*** (3292.11) | 3.094*** (3256.79) | 3.360*** (5775.06) | 3.354*** (5747.93) | 3.020*** (2684.16) | 3.006*** (2622.97) | 3.254*** (4029.03) | 3.244*** (3948.61) |
| Observations | 7722 | 7718 | 1962 | 1961 | 1924 | 1922 | 1911 | 1910 | 1925 | 1925 |

Note: z statistics in parentheses; + p<0.10, * p<0.05, ** p<0.01, *** p<0.001.

**Table S16.** Linear regression models on the percentage of money shared in the MDG

|  | (1)  Pooled model | (2)  Pooled model | (3)  Germany | (4)  Germany | (5)  Poland | (6)  Poland | (7)  Sweden | (8)  Sweden | (9)  USA | (10)  USA |
| --- | --- | --- | --- | --- | --- | --- | --- | --- | --- | --- |
|  | Objective  SES | Subjective  SES | Objective  SES | Subjective  SES | Objective  SES | Subjective  SES | Objective  SES | Subjective  SES | Objective  SES | Subjective  SES |
| Objective social status (composite, cent.) | 1.912* (5.66) |  | 1.342* (2.52) |  | 1.225+ (1.78) |  | 2.870*** (4.93) |  | 2.329*** (3.57) |  |
|  |  |  |  |  |  |  |  |  |  |  |
| Recipient: No status info | -7.716** (-6.41) | -7.559* (-5.55) | -6.689*** (-4.48) | -6.230*** (-4.20) | -5.808** (-3.21) | -5.791** (-3.23) | -6.784*** (-4.18) | -6.451*** (-4.04) | -11.40*** (-6.25) | -11.89*** (-6.62) |
|  |  |  |  |  |  |  |  |  |  |  |
| Recipient: Middle status | -4.819* (-4.24) | -4.901* (-4.27) | -3.757* (-2.54) | -3.771* (-2.57) | -2.657 (-1.46) | -2.691 (-1.49) | -7.745*** (-4.85) | -7.552*** (-4.80) | -5.835** (-3.19) | -6.402*** (-3.55) |
|  |  |  |  |  |  |  |  |  |  |  |
| Recipient: High status | -8.719* (-4.61) | -8.731* (-4.76) | -7.333*** (-4.91) | -7.012*** (-4.73) | -4.602* (-2.53) | -4.885** (-2.70) | -13.66*** (-8.55) | -13.42*** (-8.54) | -9.705*** (-5.28) | -10.05*** (-5.56) |
|  |  |  |  |  |  |  |  |  |  |  |
| Age | 0.0852 (1.78) | 0.0853 (1.59) | 0.0500 (1.58) | 0.0449 (1.44) | 0.102* (2.24) | 0.126** (2.93) | 0.0970** (2.85) | 0.0804* (2.39) | -0.0170 (-0.46) | -0.0160 (-0.45) |
|  |  |  |  |  |  |  |  |  |  |  |
| Male | 1.259 (1.45) | 0.951 (0.89) | -0.271 (-0.26) | -0.374 (-0.36) | 3.426** (2.62) | 3.598** (2.80) | -0.464 (-0.37) | -1.506 (-1.23) | 1.967 (1.52) | 1.445 (1.14) |
|  |  |  |  |  |  |  |  |  |  |  |
| Subjective social status |  | 1.934** (7.30) |  | 1.588*** (5.90) |  | 1.483*** (4.86) |  | 2.509*** (9.23) |  | 2.345*** (8.57) |
|  |  |  |  |  |  |  |  |  |  |  |
| Constant | 41.44** (7.82) | 32.03** (6.64) | 43.11*** (22.11) | 35.28*** (14.80) | 30.34*** (12.87) | 21.83*** (8.15) | 48.56*** (25.65) | 37.16*** (16.67) | 49.40*** (22.49) | 38.93*** (15.96) |
| Observations | 7722 | 7718 | 1962 | 1961 | 1924 | 1922 | 1911 | 1910 | 1925 | 1925 |
| Log likelihood | -36311.6 | -36214.5 | -8965.7 | -8946.1 | -9152.0 | -9126.7 | -8842.9 | -8807.8 | -9160.3 | -9130.5 |
| AIC | 72629.2 | 72435.1 | 17945.4 | 17906.2 | 18318.0 | 18267.3 | 17699.8 | 17629.6 | 18334.5 | 18274.9 |
| BIC | 72650.1 | 72455.9 | 17984.5 | 17945.3 | 18356.9 | 18306.2 | 17738.7 | 17668.5 | 18373.5 | 18313.8 |

Note: t statistics in parentheses; + p<0.10, * p<0.05, ** p<0.01, *** p<0.001.

**Table S17.** Linear regression models on the percentage of time kept in the TDG

|  | (11)  Pooled model | (12)  Pooled model | (13)  Germany | (14)  Germany | (15)  Poland | (16)  Poland | (17)  Sweden | (18)  Sweden | (19)  USA | (20)  USA |
| --- | --- | --- | --- | --- | --- | --- | --- | --- | --- | --- |
|  | Objective  SES | Subjective  SES | Objective  SES | Subjective  SES | Objective  SES | Subjective  SES | Objective  SES | Subjective  SES | Objective  SES | Subjective  SES |
| Objective social status (composite, cent.) | -1.482+ (-2.47) |  | -1.031+ (-1.91) |  | -0.541 (-0.71) |  | -1.558** (-2.95) |  | -3.235*** (-5.09) |  |
|  |  |  |  |  |  |  |  |  |  |  |
| Recipient: No status info | -1.178 (-1.92) | -1.304 (-2.04) | -2.683+ (-1.77) | -2.888+ (-1.91) | -0.0242 (-0.01) | -0.215 (-0.11) | -1.439 (-0.99) | -1.482 (-1.03) | -0.865 (-0.49) | -0.837 (-0.48) |
|  |  |  |  |  |  |  |  |  |  |  |
| Recipient: Middle status | -1.753 (-1.71) | -1.760 (-1.70) | -4.748** (-3.12) | -4.754** (-3.13) | -0.466 (-0.23) | -0.412 (-0.21) | -0.528 (-0.37) | -0.511 (-0.36) | -1.296 (-0.73) | -1.443 (-0.82) |
|  |  |  |  |  |  |  |  |  |  |  |
| Recipient: High status | -2.394 (-1.80) | -2.301 (-1.78) | -6.044*** (-4.04) | -5.819*** (-3.90) | -1.655 (-0.82) | -1.846 (-0.92) | -0.0423 (-0.03) | 0.250 (0.17) | -1.586 (-0.89) | -1.770 (-1.00) |
|  |  |  |  |  |  |  |  |  |  |  |
| Age | -0.0676* (-3.25) | -0.0672+ (-2.90) | -0.0458 (-1.42) | -0.0413 (-1.30) | -0.0103 (-0.20) | -0.0198 (-0.42) | -0.0783* (-2.54) | -0.0681* (-2.23) | -0.0270 (-0.75) | -0.0370 (-1.05) |
|  |  |  |  |  |  |  |  |  |  |  |
| Male | -2.054+ (-3.14) | -1.795 (-2.15) | -1.615 (-1.50) | -1.550 (-1.45) | -3.500* (-2.43) | -3.468* (-2.44) | -0.289 (-0.26) | 0.599 (0.54) | -3.126* (-2.48) | -2.478* (-1.99) |
|  |  |  |  |  |  |  |  |  |  |  |
| Subjective social status |  | -1.601** (-11.43) |  | -1.201*** (-4.37) |  | -1.620*** (-4.82) |  | -1.751*** (-7.08) |  | -2.055*** (-7.66) |
|  |  |  |  |  |  |  |  |  |  |  |
| Constant | 56.02*** (18.03) | 63.78*** (20.74) | 56.16*** (28.59) | 61.92*** (25.88) | 60.15*** (23.14) | 68.79*** (23.15) | 52.34*** (30.19) | 60.09*** (29.85) | 50.74*** (24.04) | 60.63*** (25.36) |
| Observations | 7722 | 7718 | 1962 | 1961 | 1924 | 1922 | 1911 | 1910 | 1925 | 1925 |
| Log likelihood | -36328.0 | -36254.6 | -8997.9 | -8985.7 | -9339.7 | -9315.9 | -8652.4 | -8625.8 | -9110.0 | -9093.9 |
| AIC | 72661.9 | 72515.3 | 18009.8 | 17985.5 | 18693.4 | 18645.8 | 17318.9 | 17265.7 | 18234.0 | 18201.8 |
| BIC | 72682.8 | 72536.1 | 18048.9 | 18024.5 | 18732.3 | 18684.8 | 17357.8 | 17304.6 | 18273.0 | 18240.8 |

Note: t statistics in parentheses; + p<0.10, * p<0.05, ** p<0.01, *** p<0.001.

**Table S18.** Cragg Hurdle Models on the percentage of money shared in the MDG, including recipient interaction effects

|  | (1)  Pooled model | (2)  Pooled model | (3)  Germany | (4)  Germany | (5)  Poland | (6)  Poland | (7)  Sweden | (8)  Sweden | (9)  USA | (10)  USA |
| --- | --- | --- | --- | --- | --- | --- | --- | --- | --- | --- |
|  | Objective  SES | Subjective  SES | Objective  SES | Subjective SES | Objective SES | Subjective SES | Objective  SES | Subjective  SES | Objective  SES | Subjective SES |
| Percentage given MDG |  |  |  |  |  |  |  |  |  |  |
| Social status (composite, cent.) | 4.448*** (10.13) |  | 5.272*** (4.72) |  | 2.864 (1.41) |  | 4.111*** (3.75) |  | 5.161*** (3.56) |  |
|  |  |  |  |  |  |  |  |  |  |  |
| Subjective social status |  | 2.881*** (16.36) |  | 2.551*** (4.65) |  | 3.001** (3.21) |  | 3.010*** (5.47) |  | 3.547*** (5.57) |
|  |  |  |  |  |  |  |  |  |  |  |
| Recipient: No status info | -6.599*** (-4.44) | -1.686 (-0.63) | -4.977** (-3.20) | -0.603 (-0.14) | -5.003+ (-1.70) | 1.381 (0.18) | -5.776*** (-3.65) | 1.820 (0.44) | -11.18*** (-5.07) | -7.787 (-1.53) |
|  |  |  |  |  |  |  |  |  |  |  |
| Recipient: Middle status | -5.029*** (-7.30) | -1.445 (-1.02) | -3.850* (-2.55) | 1.519 (0.35) | -4.187 (-1.45) | -3.641 (-0.48) | -6.268*** (-4.01) | -4.447 (-1.03) | -6.385** (-2.98) | 0.812 (0.17) |
|  |  |  |  |  |  |  |  |  |  |  |
| Recipient: High status | -8.962*** (-5.50) | -5.533* (-2.57) | -6.753*** (-4.35) | 0.00946 (0.00) | -5.994* (-2.03) | -6.196 (-0.78) | -12.90*** (-8.09) | -6.314 (-1.43) | -9.930*** (-4.52) | -8.920+ (-1.75) |
|  |  |  |  |  |  |  |  |  |  |  |
| Recipient: No status info * Social status (composite, cent.) | -1.958* (-2.40) |  | -3.196* (-2.02) |  | -3.382 (-1.16) |  | -1.122 (-0.70) |  | 0.259 (0.12) |  |
|  |  |  |  |  |  |  |  |  |  |  |
| Recipient: Middle status * Social status (composite, cent.) | -3.163** (-3.13) |  | -4.802** (-3.07) |  | -0.589 (-0.21) |  | -1.766 (-1.14) |  | -4.956* (-2.31) |  |
|  |  |  |  |  |  |  |  |  |  |  |
| Recipient: High status * Social status (composite, cent.) | -3.046+ (-1.88) |  | -7.147*** (-4.50) |  | 0.0357 (0.01) |  | -2.265 (-1.38) |  | -1.280 (-0.59) |  |
|  |  |  |  |  |  |  |  |  |  |  |
| Recipient: No status info * Subjective social status |  | -0.970*** (-3.62) |  | -0.784 (-0.98) |  | -1.298 (-0.95) |  | -1.464+ (-1.94) |  | -0.841 (-0.89) |
|  |  |  |  |  |  |  |  |  |  |  |
| Recipient: Middle status * Subjective social status |  | -0.755* (-2.51) |  | -1.075 (-1.36) |  | -0.133 (-0.10) |  | -0.361 (-0.47) |  | -1.606+ (-1.76) |
|  |  |  |  |  |  |  |  |  |  |  |
| Recipient: High status * Subjective social status |  | -0.715+ (-1.96) |  | -1.312 (-1.63) |  | -0.0540 (-0.04) |  | -1.283 (-1.62) |  | -0.303 (-0.32) |
|  |  |  |  |  |  |  |  |  |  |  |
| Age | 0.00862 (0.18) | 0.00758 (0.14) | 0.00567 (0.17) | -0.00442 (-0.14) | 0.0669 (0.90) | 0.0982 (1.43) | 0.0276 (0.82) | 0.0165 (0.50) | -0.109* (-2.45) | -0.1000* (-2.32) |
|  |  |  |  |  |  |  |  |  |  |  |
| Male | 2.946* (2.28) | 2.548+ (1.80) | 0.884 (0.80) | 0.761 (0.69) | 6.766** (3.16) | 7.100*** (3.42) | 0.788 (0.64) | -0.0312 (-0.03) | 4.964** (3.16) | 3.990** (2.61) |
|  |  |  |  |  |  |  |  |  |  |  |
| Constant | 46.72*** (8.88) | 32.78*** (6.03) | 46.70*** (23.11) | 34.32*** (9.81) | 29.28*** (7.30) | 13.12* (2.12) | 53.85*** (28.75) | 39.52*** (11.72) | 53.79*** (20.56) | 37.56*** (9.59) |
| Given MDG > 0 |  |  |  |  |  |  |  |  |  |  |
| Social status (composite, cent.) | 0.0675+ (1.67) |  | -0.0961 (-1.07) |  | 0.0842 (1.19) |  | 0.176+ (1.89) |  | 0.0846 (1.03) |  |
|  |  |  |  |  |  |  |  |  |  |  |
| Subjective social status |  | 0.0315+ (1.87) |  | -0.0148 (-0.34) |  | 0.0127 (0.42) |  | 0.0745+ (1.74) |  | 0.0671+ (1.88) |
|  |  |  |  |  |  |  |  |  |  |  |
| Recipient: No status info | -0.305*** (-9.80) | -0.324*** (-5.76) | -0.362** (-3.08) | -0.511 (-1.61) | -0.272** (-2.96) | -0.304 (-1.32) | -0.252* (-1.96) | -0.154 (-0.52) | -0.395*** (-3.53) | -0.374 (-1.64) |
|  |  |  |  |  |  |  |  |  |  |  |
| Recipient: Middle status | -0.114* (-2.00) | -0.163 (-1.37) | -0.0816 (-0.65) | -0.403 (-1.21) | -0.0434 (-0.45) | -0.0421 (-0.18) | -0.315* (-2.52) | -0.451 (-1.56) | -0.139 (-1.18) | 0.0677 (0.28) |
|  |  |  |  |  |  |  |  |  |  |  |
| Recipient: High status | -0.244*** (-4.39) | -0.273** (-3.01) | -0.254* (-2.10) | -0.549+ (-1.71) | -0.146 (-1.55) | -0.174 (-0.73) | -0.380** (-3.07) | -0.483+ (-1.65) | -0.307** (-2.69) | -0.131 (-0.55) |
|  |  |  |  |  |  |  |  |  |  |  |
| Recipient: No status info * Social status (composite, cent.) | -0.0832+ (-1.79) |  | 0.0936 (0.81) |  | -0.0807 (-0.85) |  | -0.167 (-1.30) |  | -0.142 (-1.30) |  |
|  |  |  |  |  |  |  |  |  |  |  |
| Recipient: Middle status * Social status (composite, cent.) | -0.0726 (-1.35) |  | 0.136 (1.09) |  | -0.143 (-1.46) |  | -0.0767 (-0.62) |  | -0.135 (-1.18) |  |
|  |  |  |  |  |  |  |  |  |  |  |
| Recipient: High status * Social status (composite, cent.) | -0.0446 (-0.55) |  | 0.238* (2.00) |  | -0.0341 (-0.35) |  | -0.233+ (-1.88) |  | -0.147 (-1.32) |  |
|  |  |  |  |  |  |  |  |  |  |  |
| Recipient: No status info * Subjective social status |  | 0.00487 (0.72) |  | 0.0322 (0.55) |  | 0.00727 (0.17) |  | -0.0154 (-0.27) |  | -0.00780 (-0.17) |
|  |  |  |  |  |  |  |  |  |  |  |
| Recipient: Middle status * Subjective social status |  | 0.0105 (0.53) |  | 0.0662 (1.08) |  | 0.000586 (0.01) |  | 0.0336 (0.60) |  | -0.0481 (-1.00) |
|  |  |  |  |  |  |  |  |  |  |  |
| Recipient: High status * Subjective social status |  | 0.00683 (0.43) |  | 0.0618 (1.05) |  | 0.00550 (0.12) |  | 0.0265 (0.47) |  | -0.0424 (-0.88) |
|  |  |  |  |  |  |  |  |  |  |  |
| Age | 0.00892*** (8.98) | 0.00892*** (8.37) | 0.00704** (2.83) | 0.00711** (2.88) | 0.00599* (2.55) | 0.00636** (2.84) | 0.0104*** (4.03) | 0.00943*** (3.62) | 0.00654** (2.94) | 0.00604** (2.73) |
|  |  |  |  |  |  |  |  |  |  |  |
| Male | -0.0852* (-2.03) | -0.0947* (-2.04) | -0.141+ (-1.72) | -0.149+ (-1.81) | 0.0103 (0.16) | 0.00945 (0.14) | -0.136 (-1.49) | -0.191* (-2.05) | -0.158* (-2.05) | -0.150+ (-1.96) |
|  |  |  |  |  |  |  |  |  |  |  |
| Constant | 0.982*** (5.19) | 0.832*** (5.39) | 1.292*** (8.41) | 1.357*** (4.94) | 0.704*** (5.86) | 0.620** (3.24) | 1.198*** (8.44) | 0.889*** (3.58) | 1.235*** (9.19) | 0.959*** (4.91) |
| lnsigma |  |  |  |  |  |  |  |  |  |  |
| Constant | 3.265*** (36.47) | 3.251*** (36.68) | 3.092^***^ (151.00) | 3.082^***^ (151.25) | 3.479^***^ (109.53) | 3.461^***^ (110.54) | 3.352^***^ (140.24) | 3.333^***^ (141.24) | 3.113^***^ (157.84) | 3.100^***^ (158.00) |
| Observations | 7722 | 7718 | 1962 | 1961 | 1924 | 1922 | 1911 | 1910 | 1925 | 1925 |
| Log likelihood | -33621.0 | -33527.0 | -8492.0 | -8483.4 | -7978.1 | -7952.4 | -8356.3 | -8321.6 | -8529.4 | -8503.3 |
| AIC | 67250.0 | 67062.0 | 17026.1 | 17008.8 | 15998.3 | 15946.7 | 16754.6 | 16685.3 | 17100.9 | 17048.6 |
| BIC | 67277.8 | 67089.8 | 17143.3 | 17126.0 | 16115.1 | 16063.5 | 16871.3 | 16801.9 | 17217.7 | 17165.5 |

Note: z statistics in parentheses; + p<0.10, * p<0.05, ** p<0.01, *** p<0.001.

**Table S19.** Cragg Hurdle Models on the percentage of time kept in the TDG, including recipient interaction effects

|  | (11)  Pooled model | (12)  Pooled model | (13)  Germany | (14) Germany | (15)  Poland | (16) Poland | (17)  Sweden | (18)  Sweden | (19)  USA | (20)  USA |
| --- | --- | --- | --- | --- | --- | --- | --- | --- | --- | --- |
|  | Objective  SES | Subjective  SES | Objective  SES | Subjective SES | Objective  SES | Subjective SES | Objective  SES | Subjective  SES | Objective  SES | Subjective SES |
| Percentage kept TDG |  |  |  |  |  |  |  |  |  |  |
| Social status (composite, cent.) | -1.654** (-3.10) |  | -1.659 (-1.55) |  | -1.389 (-0.93) |  | -1.346 (-1.36) |  | -3.408* (-2.52) |  |
|  |  |  |  |  |  |  |  |  |  |  |
| Subjective social status |  | -1.731*** (-9.12) |  | -2.301*** (-4.24) |  | -1.682* (-2.38) |  | -1.869*** (-4.15) |  | -1.588** (-2.81) |
|  |  |  |  |  |  |  |  |  |  |  |
| Recipient: No status info | 0.812 (1.14) | -0.230 (-0.09) | -0.986 (-0.66) | -8.219* (-2.01) | 1.655 (0.82) | 0.903 (0.17) | 1.181 (0.84) | -0.366 (-0.11) | 0.809 (0.43) | 4.126 (0.99) |
|  |  |  |  |  |  |  |  |  |  |  |
| Recipient: Middle status | -1.270 (-1.08) | -1.192 (-0.55) | -3.795* (-2.53) | -7.223+ (-1.71) | -1.513 (-0.75) | -3.638 (-0.68) | 1.640 (1.19) | 1.181 (0.34) | -1.727 (-0.92) | 2.620 (0.63) |
|  |  |  |  |  |  |  |  |  |  |  |
| Recipient: High status | -2.391 (-1.60) | -2.519 (-0.80) | -5.727*** (-3.88) | -12.17** (-3.00) | -2.230 (-1.09) | -3.346 (-0.64) | 0.953 (0.69) | 3.367 (0.92) | -2.342 (-1.24) | -0.741 (-0.18) |
|  |  |  |  |  |  |  |  |  |  |  |
| Recipient: No status info * Social status (composite, cent.) | -0.0875 (-0.40) |  | -0.0215 (-0.01) |  | -0.371 (-0.18) |  | 0.415 (0.29) |  | 0.109 (0.06) |  |
|  |  |  |  |  |  |  |  |  |  |  |
| Recipient: Middle status * Social status (composite, cent.) | 0.262 (0.34) |  | 0.342 (0.22) |  | 2.145 (1.04) |  | -1.513 (-1.08) |  | 1.225 (0.66) |  |
|  |  |  |  |  |  |  |  |  |  |  |
| Recipient: High status * Social status (composite, cent.) | -0.416 (-0.89) |  | 0.526 (0.35) |  | 0.415 (0.20) |  | -1.244 (-0.88) |  | -0.174 (-0.09) |  |
|  |  |  |  |  |  |  |  |  |  |  |
| Recipient: No status info * Subjective social status |  | 0.179 (0.41) |  | 1.403+ (1.81) |  | 0.120 (0.12) |  | 0.307 (0.48) |  | -0.753 (-0.93) |
|  |  |  |  |  |  |  |  |  |  |  |
| Recipient: Middle status * Subjective social status |  | -0.0126 (-0.04) |  | 0.679 (0.86) |  | 0.450 (0.45) |  | 0.0934 (0.14) |  | -0.989 (-1.20) |
|  |  |  |  |  |  |  |  |  |  |  |
| Recipient: High status * Subjective social status |  | 0.0485 (0.13) |  | 1.318+ (1.76) |  | 0.219 (0.22) |  | -0.400 (-0.59) |  | -0.399 (-0.49) |
|  |  |  |  |  |  |  |  |  |  |  |
| Age | -0.106*** (-3.89) | -0.105*** (-3.36) | -0.0491 (-1.55) | -0.0421 (-1.34) | -0.0391 (-0.78) | -0.0524 (-1.10) | -0.117*** (-3.93) | -0.104*** (-3.53) | -0.0611 (-1.63) | -0.0701+ (-1.91) |
|  |  |  |  |  |  |  |  |  |  |  |
| Male | -0.708 (-0.89) | -0.438 (-0.44) | -0.0152 (-0.01) | 0.0897 (0.08) | -2.806+ (-1.93) | -2.893* (-2.01) | 1.239 (1.15) | 2.048+ (1.91) | -1.738 (-1.30) | -1.027 (-0.78) |
|  |  |  |  |  |  |  |  |  |  |  |
| Constant | 59.08*** (17.05) | 67.42*** (18.09) | 56.73*** (29.35) | 67.89*** (20.50) | 64.48*** (24.74) | 73.44*** (17.32) | 54.27*** (32.70) | 62.45*** (23.44) | 54.62*** (24.71) | 62.33*** (18.51) |
| Kept TDG > 0 |  |  |  |  |  |  |  |  |  |  |
| Social status (composite, cent.) | -0.0522 (-1.07) |  | 0.0588 (0.50) |  | 0.00429 (0.05) |  | -0.0561 (-0.44) |  | -0.145+ (-1.82) |  |
|  |  |  |  |  |  |  |  |  |  |  |
| Subjective social status |  | -0.0276 (-1.03) |  | 0.113+ (1.96) |  | -0.0343 (-0.85) |  | -0.141* (-2.12) |  | -0.0443 (-1.33) |
|  |  |  |  |  |  |  |  |  |  |  |
| Recipient: No status info | -0.254** (-3.22) | -0.194 (-1.11) | -0.352* (-2.40) | 0.0573 (0.16) | -0.143 (-1.23) | -0.291 (-0.97) | -0.531*** (-3.51) | -1.085* (-2.30) | -0.157 (-1.42) | 0.0875 (0.34) |
|  |  |  |  |  |  |  |  |  |  |  |
| Recipient: Middle status | -0.108 (-0.92) | -0.123 (-0.45) | -0.302* (-2.03) | 0.285 (0.75) | 0.106 (0.84) | 0.636+ (1.77) | -0.449** (-2.95) | -1.288** (-2.75) | 0.0165 (0.14) | -0.159 (-0.61) |
|  |  |  |  |  |  |  |  |  |  |  |
| Recipient: High status | -0.0584 (-0.86) | -0.0575 (-0.28) | -0.213 (-1.42) | 0.590 (1.48) | 0.0484 (0.39) | 0.0512 (0.16) | -0.254 (-1.58) | -1.208* (-2.43) | 0.0364 (0.31) | 0.0534 (0.20) |
|  |  |  |  |  |  |  |  |  |  |  |
| Recipient: No status info * Social status (composite, cent.) | 0.0689** (2.77) |  | 0.0837 (0.56) |  | -0.000631 (-0.01) |  | 0.0944 (0.60) |  | 0.0699 (0.63) |  |
|  |  |  |  |  |  |  |  |  |  |  |
| Recipient: Middle status * Social status (composite, cent.) | 0.0146 (0.27) |  | -0.0971 (-0.64) |  | -0.100 (-0.78) |  | 0.139 (0.89) |  | 0.0461 (0.41) |  |
|  |  |  |  |  |  |  |  |  |  |  |
| Recipient: High status * Social status (composite, cent.) | 0.0909** (2.85) |  | -0.0382 (-0.25) |  | 0.145 (1.14) |  | 0.0621 (0.38) |  | 0.0991 (0.84) |  |
|  |  |  |  |  |  |  |  |  |  |  |
| Recipient: No status info * Subjective social status |  | -0.0122 (-0.47) |  | -0.0916 (-1.25) |  | 0.0281 (0.52) |  | 0.0965 (1.24) |  | -0.0458 (-1.00) |
|  |  |  |  |  |  |  |  |  |  |  |
| Recipient: Middle status * Subjective social status |  | 0.00222 (0.05) |  | -0.126+ (-1.68) |  | -0.0965 (-1.60) |  | 0.153* (1.97) |  | 0.0366 (0.76) |
|  |  |  |  |  |  |  |  |  |  |  |
| Recipient: High status * Subjective social status |  | -0.000383 (-0.01) |  | -0.165* (-2.18) |  | -0.00825 (-0.15) |  | 0.175* (2.10) |  | -0.00277 (-0.06) |
|  |  |  |  |  |  |  |  |  |  |  |
| Age | 0.00333*** (4.06) | 0.00335*** (3.72) | -0.000501 (-0.17) | -0.000882 (-0.30) | 0.00299 (0.96) | 0.00295 (0.98) | 0.00440 (1.51) | 0.00451 (1.53) | 0.00275 (1.17) | 0.00244 (1.04) |
|  |  |  |  |  |  |  |  |  |  |  |
| Male | -0.212*** (-5.57) | -0.206*** (-5.39) | -0.331*** (-3.29) | -0.325** (-3.23) | -0.131 (-1.51) | -0.127 (-1.47) | -0.266* (-2.47) | -0.239* (-2.20) | -0.208* (-2.58) | -0.191* (-2.36) |
|  |  |  |  |  |  |  |  |  |  |  |
| Constant | 1.569*** (9.54) | 1.706*** (11.93) | 2.103*** (10.87) | 1.596*** (5.07) | 1.388*** (8.75) | 1.579*** (6.24) | 1.901*** (10.48) | 2.664*** (6.18) | 1.340*** (9.75) | 1.553*** (7.27) |
| lnsigma |  |  |  |  |  |  |  |  |  |  |
| Constant | 3.221*** (37.25) | 3.213*** (36.53) | 3.100*** (167.28) | 3.093*** (167.22) | 3.359*** (169.24) | 3.354*** (169.34) | 3.019*** (163.89) | 3.005*** (163.72) | 3.254*** (155.05) | 3.243*** (155.37) |
| Observations | 7722 | 7718 | 1962 | 1961 | 1924 | 1922 | 1911 | 1910 | 1925 | 1925 |
| Log likelihood | -34843.2 | -34775.4 | -8727.8 | -8709.9 | -8895.7 | -8879.4 | -8361.6 | -8331.2 | -8588.3 | -8569.2 |
| AIC | 69694.4 | 69558.8 | 17497.6 | 17461.8 | 17833.4 | 17800.8 | 16765.2 | 16704.3 | 17218.7 | 17180.5 |
| BIC | 69722.2 | 69586.6 | 17614.8 | 17579.0 | 17950.2 | 17917.6 | 16881.8 | 16821.0 | 17335.5 | 17297.3 |

Note: z statistics in parentheses; + p<0.10, * p<0.05, ** p<0.01, *** p<0.001.
